# Artemether as a modulator of EMT in colorectal cancer: enhancing radiosensitivity and reversing chemo-radiation resistance

**DOI:** 10.1186/s12876-026-04653-4

**Published:** 2026-02-04

**Authors:** Lv Ge, Shan Liu, Shenglan Yu, Ming Li, Wanni Zhang, Chunmao Xie, Zhuo Gao, Sijia Tang, Minqi Xiao, Tao Zou, Yongxin Jiang, Hu Lu

**Affiliations:** 1 Yueyang Hospital of Traditional Chinese Medicine, Yueyang, China; 2 The Third Affiliated Hospital of Kunming Medical University, Kunming, China

**Keywords:** Artemether, Colorectal cancer, Epithelial-mesenchymal transition, Radiosensitivity, Chemo-radiation resistance

## Abstract

**Background:**

The efficacy of conventional chemoradiotherapy for colorectal cancer is often limited by resistance, with epithelial-mesenchymal transition being a key mechanism. Although the artemisinin derivative artemether (ARE) has shown antitumor potential, it remains unclear whether it can enhance radiosensitivity and reverse chemo-radiation resistance in colorectal cancer by regulating EMT. This study aimed to investigate the radiosensitizing and resistance-reversing effects of ARE on human colorectal cancer xenografts in nude mice and to elucidate the underlying mechanism related to EMT regulation.

**Methods:**

A nude mouse xenograft model using human colorectal cancer HCT116 and HCT116-chemo-radiation resistant (HCT116-CRR) cells was established.

**Results:**

ARE combined with radiotherapy suppressed tumor growth in nude mice and induced cell death via necrosis and apoptosis. After ARE combined with radiotherapy, β-Catenin was increased in human colorectal cancer HCT116 cells implanted in nude mice, while Vimentin was decreased. In HCT116-CRR cells transplanted into nude mice, the E-cadherin and β-Catenin were upregulated, whereas N-Cadherin, Vimentin, Snail, Slug, and Twist were downregulated. ARE effectively significantly enhanced radiosensitivity and reversed chemo-radiation resistance by suppressing EMT.

**Conclusions:**

These findings provide both mechanistic insights and experimental validation for the potential application of ARE as a radiosensitizer in colorectal cancer radiotherapy.

**Supplementary Information:**

The online version contains supplementary material available at 10.1186/s12876-026-04653-4.

## Introduction

Colorectal cancer (CRC) is a highly prevalent malignancy of the digestive system with the increasing incidence and mortality [1–3]. With 517,100 new cases in 2022 in China, CRC was the second most prevalent type of cancers among all malignant tumors [4]. The incidence rate among young people is progressively increasing, particularly in developed countries [5]. Current treatment strategies for CRC primarily involve surgery, chemotherapy, and radiotherapy [6–9]. Neoadjuvant concurrent chemoradiotherapy based on 5-fluorouracil (5-Fu) is a standardized treatment regimen recommended by the NCCN Clinical Practice Guidelines for rectal cancer [10]. However, as the use of neoadjuvant chemoradiotherapy becomes increasingly widespread, resistance to concurrent chemoradiotherapy is also becoming more prominent. According to literature reports, the complete pathological remission rate of patients was only 8% to 29% after neoadjuvant chemoradiotherapy, and some patients still failed to benefit from the treatment [11]. With the increasingly widespread application of chemoradiotherapy, addressing the issue of residual cancer cell resistance is urgent. Researchers have been exploring the mechanisms of chemoradiotherapy resistance. Recently, more attention has been focused on epithelial-mesenchymal transition (EMT), which is a reversible process that operates in both physiological and pathological contexts [12, 13]. It is characterized by the gradual loss of epithelial features, such as cell-cell adhesion and apical-basal polarity, along with the concurrent acquisition of mesenchymal phenotypes [14]. Its pathological activation enhances carcinoma aggressiveness by increasing cellular motility and invasiveness. EMT imparts therapeutic resistance and promotes immune evasion mechanisms in malignant cells [15, 16]. Additionally, it has been demonstrated that multiple factors influence the sensitization of tumors to radiotherapy including cell cycle arrest, DNA damage repair, miRNA expression variations, tumor microenvironment dynamics, hypoxia, and autophagy regulation [17]. Therefore, finding a low toxicity and efficient drug to reverse their resistance and enhance radiosensitivity has become a key issue affecting the efficacy of neoadjuvant therapy for CRC patients.

Natural products have garnered significant attention in anticancer research due to their multi-target effects and favorable safety profiles. Artemisinin and its derivatives have demonstrated significant anti-inflammatory, antifibrotic, and antitumor properties [18–20]. Artemisinin, a sesquiterpene lactone isolated from *Artemisia annua*, exhibits limited clinical utility owing to poor solubility and bioavailability [21]. Artemether (ARE) is the methyl ether derivative of dihydroartemisinic acid, which serves as the active metabolite of artemisinin. This lipophilic derivative exhibits the enhanced hydrophobic characteristic, demonstrating relative stability in biological fluids and ensuring rapid and consistent oral bioavailability [22]. ARE exhibits dose- and time-dependent cytotoxicity against CRC cells [23]. Preclinical studies with CRC xenograft models have established its therapeutic potential and optimized in vitro radiation parameters [24]. However, direct in vivo and in vitro evidence systematically demonstrating whether ARE can reverse chemo-radiation resistance and enhance radiosensitivity in CRC by regulating the EMT pathway remains lacking.

Therefore, this study aims to systematically evaluate the radiosensitivity and chemo-radiation resistance of ARE in CRC models, particularly its relationship with EMT regulation. By investigating the role of ARE in enhancing radiosensitivity and reversing chemo-radiation resistance *via* EMT modulation, this research aims to establish a mechanistic foundation for improving radiotherapy outcomes in CRC patients.

## Materials and methods

### Chemicals and reagents

Artemether (ARE, Batch No.:20211228-01) was provided as a lyophilized powder by Kunming Pharmaceutical Group Co., Ltd. DMEM high-glucose medium (Catalog No. C11995500BT), PBS buffer (Catalog No. C10010500BT), and penicillin-streptomycin solution (Catalog No. 15140148) were purchased from Gibco (USA). Hematoxylin Staining Solution (Catalog No. C0107), Hematoxylin-Eosin (H&E) Staining Kit (Catalog No. C0105S), 4% paraformaldehyde fixative solution (Catalog No. P0099-3 L), and sodium citrate antigen retrieval solution (Catalog No. P0081) were obtained from Beyotime Institute of Biotechnology (China). Xylene (Catalog No. 016371) and ethanol (Catalog No. 033361) were purchased from Thermo Fisher Scientific (USA). The rabbit-specific HRP/DAB (ABC) detection kit (Catalog No. ab64261) was obtained from Abcam (USA). The Annexin V-FITC/PI Apoptosis Detection Kit (Catalog No. 556547) was from BD Biosciences (USA); Triton X-100 was from Sigma-Aldrich (USA). TRIzol™ Reagent (Catalog No. 15596026) was purchased from Invitrogen (USA); the Transcriptor First Strand cDNA Synthesis Kit (Catalog No. 04897030001) was from Roche (Switzerland); and the TB Green^®^ Premix Ex Taq™ II kit (Catalog No. RR820A) was from Takara (Japan). The BCA protein quantification kit (Catalog No. P0012) was obtained from Beyotime Institute of Biotechnology (China). RIPA lysis buffer (Catalog No. CBW0011), PMSF (100 mM, Catalog No. CBW0017), the SDS-PAGE gel preparation kit (Catalog No. CBW0001–CBW0010), and the BCA protein quantification detection kit (Catalog No. CBW0020) were purchased from Wuhan Cobioer Biosciences Co., Ltd. (China). Antibodies against E-cadherin (Catalog No. BF0219), Snail (Catalog No. AF6032), Twist (Catalog No. AF4009), β-catenin (Catalog No. BF8016), N-Cadherin (Catalog No. AF5239), Vimentin (Catalog No. AF7013), Slug (Catalog No. AF4002), GAPDH (Catalog No. AF7021), Anti-mouse IgG (Catalog No. S0002) were all purchased from Affinity Biosciences (China). All primary antibodies were used at an optimized working dilution of 1:1000.

### Preparation of ARE working solution and vehicle control

The ARE working solution was prepared as follows: 60 mg of ARE powder was accurately weighed and dissolved in 5 mL of normal saline containing 2% (v/v) Tween-80 with thorough vortexing. The volume was then adjusted to 12 mL with normal saline to obtain a stock solution at a concentration of 5 mg/mL. This stock solution was sterilized by filtration through a 0.22 μm sterile filter, aliquoted, stored at 4 °C protected from light, and used within 2 weeks. The vehicle control for the control group consisted of normal saline containing an identical concentration of Tween-80 (2%, v/v), which was prepared, filtered, and stored under the same conditions as the ARE working solution.

### Animals and colon cancer cell culture

A total of 40 specific pathogen-free (SPF) grade male BALB/c-nu nude mice (26–30 days old) were purchased from Hunan Silaike Jingda Experimental Animal Co., Ltd. All mice were kept in plastic cages under standard conditions of 22℃ ± 2, with a 12:12 h light-dark cycle, and had unlimited access to food and water. The experiments were approved by (Ethical approval number HTDW-202310001).

HCT116 cell line was obtained from the Kunming Cell Bank, Chinese Academy of Sciences. HCT116-CRR cell line was established by our research group. HCT116 and HCT116-CRR cells were cultured in DMEM medium, containing 10% of fetal bovine serum (*v/v*) and 1% penicillin-streptomycin (*v/v*) at 37℃ with 5% CO_2_ in a humidified atmosphere.

### Establishment and validation of chemo‑radioresistant HCT116‑CRR cell line

The HCT116‑CRR cell line was established according to the method previously established by our research group [25]. Briefly, parental HCT116 cells were subjected to repeated cycles of exposure to 5‑fluorouracil (5‑FU, 10 µmol/L) combined with a single dose of 4 Gy irradiation (6 MeV X‑rays), followed by incubation in drug‑containing medium for 24 h and then recovery in drug‑free medium. This combined treatment and recovery procedure was repeated for 9 cycles, ultimately yielding a stable chemo‑radioresistant cell line designated HCT116‑CRR. To validate the resistance, clonogenic assays were performed. The results showed that compared with parental HCT116 cells, HCT116‑CRR cells exhibited significantly reduced sensitivity to the combination of 5‑FU and radiation, as evidenced by significant increases in both the D₀ value (2.79 Gy vs. 2.30 Gy) and the SF₂ value (0.72 vs. 0.59). Furthermore, distinct morphological alterations were observed in HCT116‑CRR cells, including elongation, deformation, pseudopodia formation, and a more dispersed growth pattern compared to the densely packed parental cells. Together, these functional and morphological findings confirm that the HCT116‑CRR cell line possesses a stable acquired chemo‑radioresistant phenotype.

### Establishment and treatment of colon cancer cell xenograft models in vivo

The tumor xenograft in nude mice was created using a subcutaneous injection of HCT116 colon cancer cells. A single-cell suspension of HCT116 cells was aspirated into a 1 mL microliter syringe and subcutaneously inoculated into the dorsal region and right femoral base of five aseptically prepared BALB/c-nu nude mice (0.2 mL per site). The mice were subsequently maintained under SPF conditions for monitoring. When primary tumors reached 0.5 × 0.5 × 0.5 cm³, tumor-bearing mice were euthanized by cervical dislocation. Tumors were aseptically excised, minced into 2 mm³ fragments, and transplanted into the dorsal region of 25 disinfected BALB/c-nu nude mice using an18-gauge trocar. Inoculation sites were disinfected, and mice were housed under SPF conditions. When tumor volumes in HCT116 cell-bearing nude mice reached 0.5 × 0.5 × 0.5 cm³, tumor-bearing nude mice were randomly allocated into four experimental groups (*n* = 5 per group) using a random number table. A subcutaneous transplant tumor model of HCT116-CRR cells in nude mice was established using the above same method. The experimental groups and treatment regimens were as follows: Control group (Control): Injected with normal saline; Artemether group (ARE): Injected with artemether; Radiotherapy group (RT): Injected with normal saline + radiotherapy; Combination group (RT + ARE): Injected with artemether + radiotherapy. To minimize subjective bias, all tumor volume measurements during treatment, tumor weight assessments after treatment, and subsequent histopathological evaluations were performed by investigators blinded to group assignments (i.e., in a single-blinded manner).

### Treatment of colon cancer cell xenograft models in *vivo*

All tumor-bearing nude mice were treated within the same time period. Agents were administered daily via intraperitoneal injection for 14 consecutive days: the control and radiotherapy (RT) groups received normal saline (90 mg/kg), while the artemether (ARE) and combination (RT + ARE) groups received artemether (50 mg/kg).

The artemether dosing regimen (50 mg/kg) was determined with reference to the human equivalent dose (HED) conversion method recommended by the U.S. Food and Drug Administration (FDA). This dose corresponds to an HED of approximately 4.1 mg/(kg·d) (equivalent to about 246 mg daily for a 60 kg adult), which is close to the initial clinical antimalarial dosage of artemether (3.2 mg/kg), indicating that the dose used in this experiment falls within a clinically relevant range. For mice undergoing radiotherapy (RT and RT + ARE groups), a standardized procedure was followed prior to each irradiation session: anesthesia was induced by intraperitoneal injection of 3% pentobarbital sodium solution (50 mg/kg). Once surgical anesthesia depth was achieved (approximately 5 min after injection), the mice were immobilized on a dedicated animal irradiation platform. Localized irradiation was delivered using a linear accelerator generating 4 MV X-rays, with the following key parameters: source-to-surface distance (SSD) of 100 cm, customized irradiation fields to ensure complete tumor coverage, and lead shielding to protect surrounding normal tissues and the whole body. A dose of 2 Gy was administered per session, repeated every other day for a total of 5 sessions. Irradiation commenced 1 h after the intraperitoneal administration of either normal saline or artemether.

### Tumor volume and body weight measurements

During the treatment period, body weight of nude mice was monitored daily. The tumor formation rate was calculated and the growth of tumors was dynamically monitored throughout the experimental course. Tumor dimensions were measured at two-day intervals post-treatment. The longest diameter (a) and shortest diameter (b) of tumors were recorded to calculate tumor volume (V) using the formula: V (cm³) = [a (cm) × b² (cm²)]/2. The tumor volume inhibition rate was determined as [1 - (mean tumor volume of experimental group/mean tumor volume of control group)] ×100%. At day 21 post-treatment, nude mice were euthanized by cervical dislocation. Tumors were aseptically excised, rinsed with saline, and weighed. Tumor inhibition rate was calculated as: [1 - (mean tumor weight of experimental group/mean tumor weight of control group)] ×100%.

### Hematoxylin and Eosin (H&E) staining

Tumor samples were fixed in 10% formalin and embedded in paraffin. 4 μm thick tissue were stained with H&E for histopathological diagnosis. Tumor tissues were deparaffinized in xylene and rehydrated in an ethanol series. H&E staining was performed according to a standard protocol. Histological observation and imaging were taken by using a BHS microscope system (Olympus Corporation, Tokyo, Japan).

### Immunohistochemistry (IHC) and evaluation

4 μm-thick serial sections were subjected to deparaffinization in xylene and rehydrated through a graded series of ethanol. Antigen retrieval was accomplished via heat treatment in sodium citrate buffer (pH 6.0), followed by blocking of non-specific antigens. Then, E-cadherin、N-cadherin、Vimentin、Snail、Slug、Twist、β-Catenin was incubated with slices overnight at 4℃, followed by incubation with biotinylated mouse anti-polyvalent. Sections were incubated with streptavidin peroxidase for 10 min in DAB for 1–8 min, and in hematoxylin for 2 min. All steps were performed in accordance with the protocol of a rabbit specific HRP/DAB (ABC) detection IHC kit (Abcam, Cambridge, MA, USA) at room temperature. Specimens were observed under a light microscope (model BX53, Olympus, Japan) equipped with a digital camera (model DP27) for image acquisition. Representative images were captured at 100× magnification. Negative controls were established by replacing primary antibodies with phosphate-buffered saline (PBS). Positive cells were identified by cytoplasmic or nuclear brownish-yellow granules. The entire section was evaluated by combined scoring of staining intensity and percentage of positive cells: Staining intensity: 0 (no staining), 1 (light yellow), 2 (brownish-yellow), 3 (dark brown); Positive cell percentage: 0 (≤ 5%), 1 (5–25%), 2 (25–50%), 3 (50–75%), 4 (> 75%). The final score was calculated as the sum of intensity and percentage scores. Tumor positivity was classified as: Negative:0–1; Weakly positive:2–4; Strongly positive:5–7 [26].

### Flow cytometric analysis of apoptosis and necrosis in tumor tissues

The apoptosis and necrosis rates of tumor tissues in each group were detected by flow cytometry. The procedure was briefly performed as follows: Tumor tissues from each group were mechanically minced and ground in sterile saline to prepare tissue homogenates. The homogenates were centrifuged at 2000 rpm for 5 min, and the supernatant was discarded. The cell pellet was resuspended in PBS and washed once, followed by another centrifugation and removal of the supernatant. The cells were adjusted to a density of 5 × 10⁵–1 × 10⁶ cells/mL with PBS. A volume of 200 µL of the cell suspension was taken and centrifuged at 1500 rpm for 5 min, after which the supernatant was discarded. Subsequently, 195 µL of Annexin V-FITC binding buffer was added to resuspend the cells, followed by sequential addition of 5 µL of Annexin V-FITC and 10 µL of propidium iodide staining solution. After gentle mixing, the samples were incubated at room temperature in the dark for 30 min and then analyzed by flow cytometry.

### Quantitative real-time PCR (qPCR) analysis

Total RNA was extracted from tissues using TRIzol reagent (Invitrogen, USA) according to the manufacturer’s instructions. Subsequently, 1 µg of total RNA was reverse-transcribed into cDNA using the Transcriptor First Strand cDNA Synthesis Kit (Roche). qPCR was performed on the Mx3005P system (Agilent Technologies, USA) with the SYBR Premix Ex Taq II kit (Takara, China). Each 20 µL reaction mixture contained 10 µL of SYBR Premix, 0.4 µM of each forward and reverse primer, 2 µL of cDNA template, and an appropriate volume of nuclease-free water. The amplification protocol was as follows: initial denaturation at 95 °C for 30 s, followed by 40 cycles of denaturation at 95 °C for 5 s and annealing/extension at 60 °C for 30 s, with fluorescence signal acquisition at this step. A melt curve analysis was performed after amplification to confirm the specificity of the PCR products. Glyceraldehyde-3-phosphate dehydrogenase (GAPDH) was selected as the reference gene for normalization, and its expression was verified to be stable across different experimental groups. The relative expression levels of the target genes were calculated using the 2 − ΔΔCt method. The specific primer sequences used are listed in Table 1.

**Table 1:** Specific sequences of primers used in RT-PCR

Gene name	Primer	Primer sequence	Length
*Mouse-E-Cadherin*	Forward Primer	CAGTTCCGAGGTCTACACCTT	21
	Reverse Primer	TGAATCGGGAGTCTTCCGAAAA	22
*Mouse-N-Cadherin*	Forward Primer	AGGCTTCTGGTGAAATTGCAT	21
	Reverse Primer	GTCCACCTTGAAATCTGCTGG	21
*Mouse-Vimentin*	Forward Primer	CGTCCACACGCACCTACAG	19
	Reverse Primer	GGGGGATGAGGAATAGAGGCT	21
*Mouse-Snail*	Forward Primer	CACACGCTGCCTTGTGTCT	19
	Reverse Primer	GGTCAGCAAAAGCACGGTT	19
*Mouse-Slug*	Forward Primer	CAGCGAACTGGACACACACACA	20
	Reverse Primer	ATAGGGCTGTATGCTCCCGAG	21
*Mouse-Twist*	Forward Primer	GGACAAGCTGAGCAAGATTCA	21
	Reverse Primer	CGGAGAAGGCGTAGCTGAG	19
*Mouse-β-Catenin*	Forward Primer	ATGGAGCCGGACAGAAAAGC	20
	Reverse Primer	TGGGAGGTGTCAACATCTTCTT	22
*Mouse-GAPDH*	Forward Primer	AGGTCGGTGTGAACGGATTTG	21
	Reverse Primer	TGTAGACCATGTAGTTGAGGTCA	24

### Western blot analysis

Tissue proteins were extracted from protein lysates with protease inhibitors. Protein samples were subjected to SDS-PAGE for the electrophoretic separation and transferred to PVDF membranes. The membranes were blocked, followed by overnight incubation with primary antibodies (E-cadherin、N-cadherin、Vimentin、Snail、Slug、Twist、β-Catenin) at 4 °C, and subsequently treated with secondary antibodies for 1 h at room temperature. Protein detection was performing using ECL chemiluminescence (Millipore Corp, Billerica, MA, USA), and quantification was carried out with Image J software.

### Statistical analysis

All data were analyzed using GraphPad Prism 9 software and are expressed as the mean ± standard deviation (mean ± SD). The normality of the data distribution was verified by the Shapiro‑Wilk test, and the homogeneity of variances was assessed using Levene’s test. For comparisons among multiple groups, one‑way analysis of variance (one‑way ANOVA) was performed. If the results showed statistical significance, Tukey’s post‑hoc test was further employed for pairwise comparisons, which has built‑in correction for multiple comparisons to control the family‑wise error rate. A *P*‑value < 0.05 was considered statistically significant.

## Results

### Suppression of tumor growth in nude mice

**Fig. 1:**
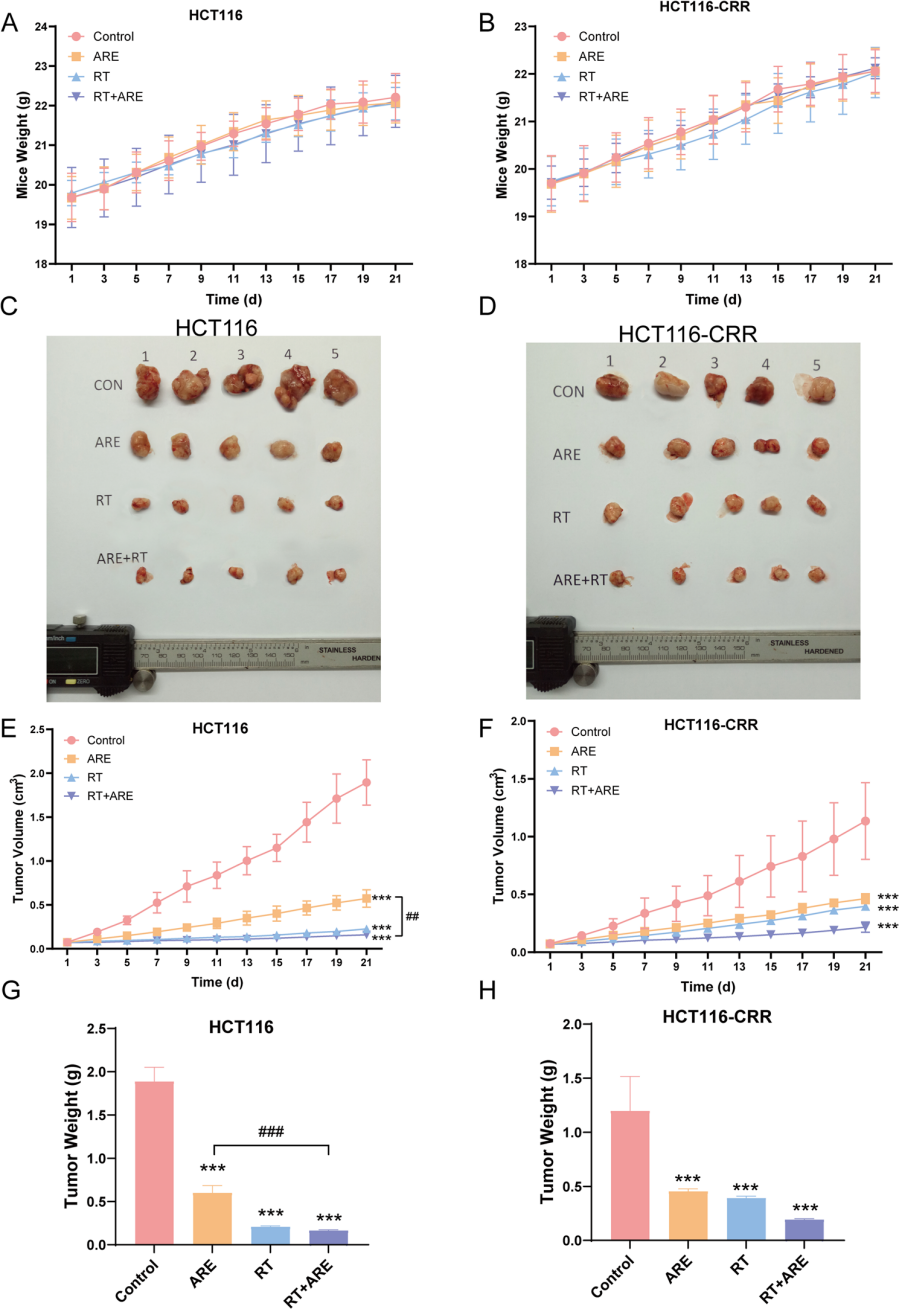
Inhibitory effect of ARE combined with radiotherapy on HCT116 and HCT116-CRR xenograft tumors in nude mice. **A**-**B** Body weight changes of tumor-bearing mice. **C**-**D** Representative photographs of xenograft tumors from each group (CON: control group). **E**-**F** Tumor volume growth curves. **G**-**H** Tumor weight at the endpoint. Data are presented as mean ± SD (*n* = 5). ^***^P < 0.001 vs. Control; ^###^*P* < 0.001 vs. RT + ARE

The HCT116 and HCT116-CRR xenograft nude mice model was established to determine the in vivo anti-cancer activity of ARE on colon cells. Body weight measurements were conducted every other day throughout the treatment. All groups demonstrated rapid weight gain during the first two weeks, followed by slower growth rates starting from the third week. No statistically significant differences in body weight were observed among groups (Fig. 1A-B).

Prior to treatment, no significant differences in tumor volume were observed among the colon cancer cell groups. Following treatment, the combination therapy group exhibited a markedly smaller tumor volume compared to other treatment groups, while all therapeutic groups demonstrated significantly reduced tumor volumes relative to the control group (*P* < 0.001, Fig. 1C-F). In HCT116 cells, the tumor inhibition rate in the combination therapy group was significantly higher than that in the ARE monotherapy group (*P* < 0.01). ARE yielded an enhancement factor (EF) of 3.20 and 2.43 for HCT116 and HCT116-CRR cell xenografts, indicating its capacity to enhance the radiosensitivity of human colorectal cancer xenografts.

The mean tumor weight of colon cancer cell-treated groups was significantly lower than that of the control group (*P* < 0.001). It is noteworthy that the radiotherapy-alone group and the radiotherapy-plus-ARE group showed comparable levels of tumor inhibition, which may suggest that the inhibitory effect had approached a plateau by the experimental endpoint. (Fig. 1G-H). These results indicated that ARE combined with RT significantly inhibited the growth of human colorectal cancer xenografts in nude mice and enhanced the therapeutic efficacy of radiotherapy.

### Cell death via necrosis and apoptosis in colon tumor tissues

**Fig. 2:**
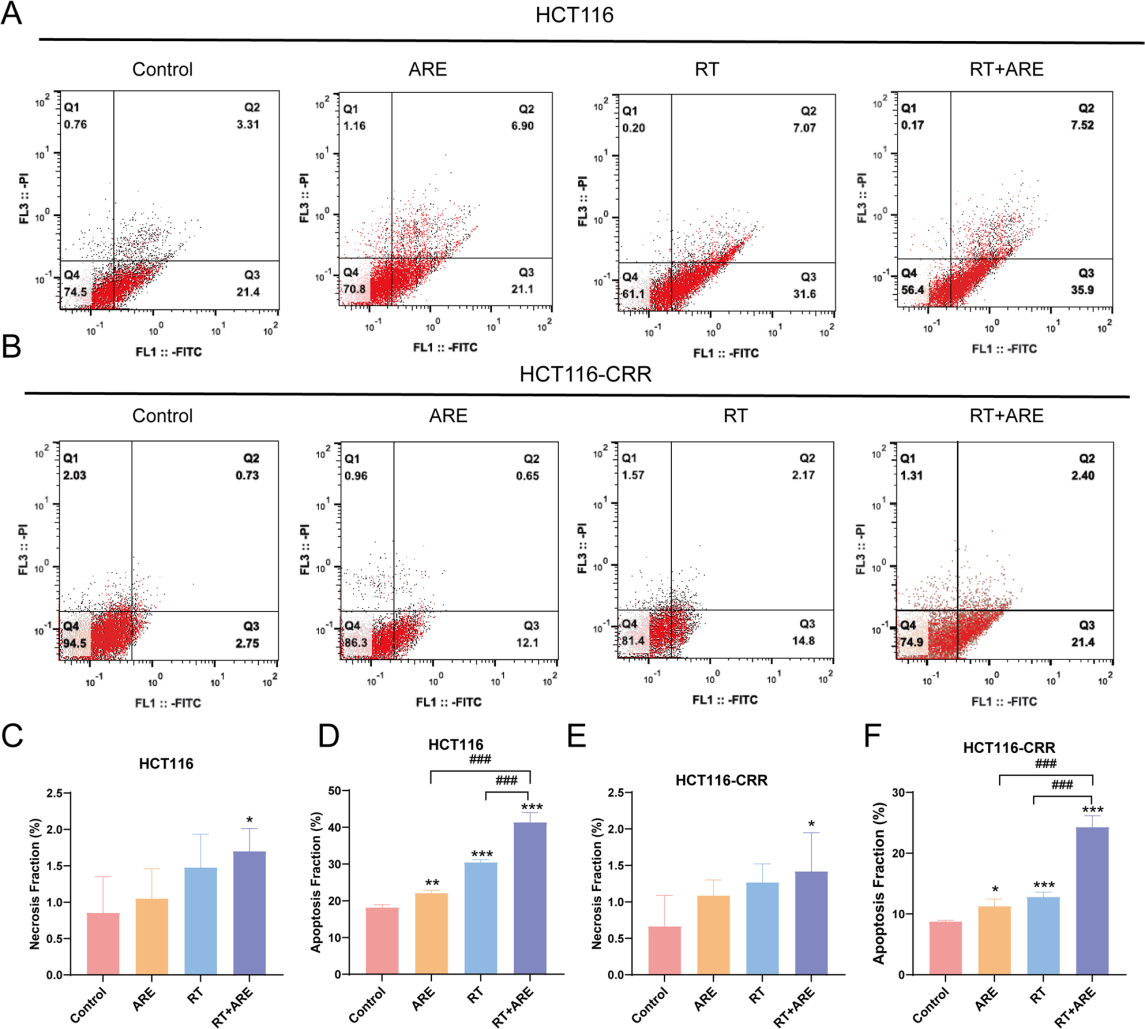
Effects of ARE combined with radiotherapy on necrosis and apoptosis in HCT116 and HCT116-CRR cells. A)Flow cytometry dot plots of HCT116 cells after various treatments (Annexin V-FITC/PI double staining). **B** Flow cytometry dot plots of HCT116-CRR cells after various treatments. **C** Quantification of necrotic cell percentage in HCT116 cells under each treatment. **D** Quantification of apoptotic cell percentage in HCT116 cells under each treatment. **E** Quantification of necrotic cell percentage in HCT116-CRR cells under each treatment. **F** Quantification of apoptotic cell percentage in HCT116-CRR cells under each treatment. Data are presented as mean ± SD (*n* = 5). ^*^P < 0.05, ^**^P < 0.01, ^***^P < 0.001 vs. Control; ^###^*P* < 0.001 vs. RT + ARE

Flow cytometry was performed to assess necrosis and apoptosis in tumor tissues derived from nude mice bearing human colorectal cancer xenografts (Fig. 2A, B). The results showed that in both HCT116 and HCT116-CRR cells, the proportion of necrosis in the RT + ARE combination treatment group was significantly higher than that in the normal control group (*P* < 0.05), while no significant changes in necrosis were observed in any of the other treatment groups (Fig. 2C, E). Regarding apoptosis, the proportion induced by the RT + ARE combination treatment was significantly higher than that in either the ARE alone or RT alone treatment groups in both HCT116 and HCT116-CRR cells (*P* < 0.001; Fig. 2D, F). These results indicate that the combination of ARE and RT effectively promotes apoptosis in the tumor tissues of nude mouse xenografts.

### Necrotic cell death in colon tumor tissues

**Fig. 3:**
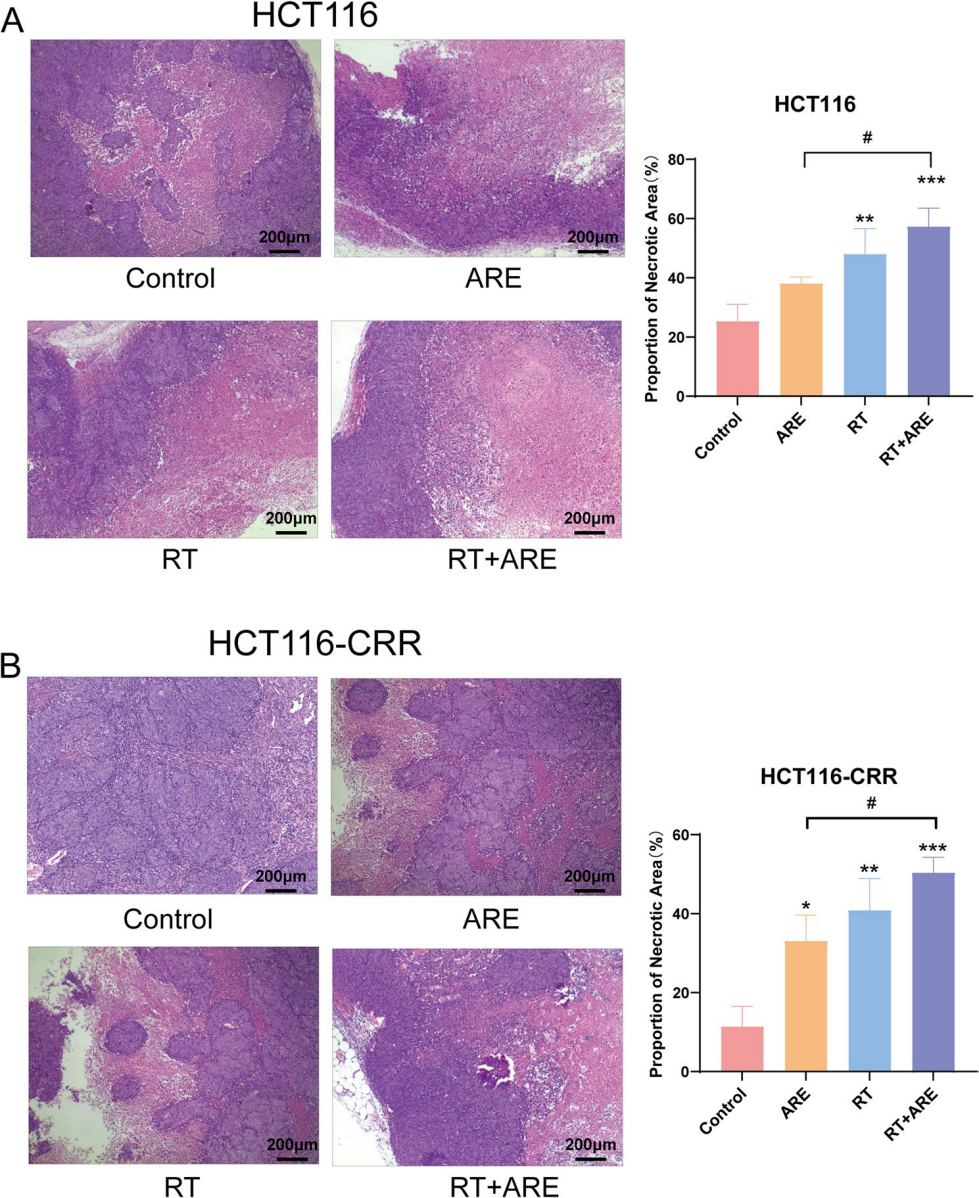
H&E staining of tumor tissues from HCT116 and HCT116-CRR xenografts and the corresponding histological necrosis scores. **A** Left panel: H&E-stained sections of HCT116 xenograft tumors (Scale bar = 200 μm); Right panel: Proportion of Necrotic Area for HCT116 xenografts. **B** Left panel: H&E-stained sections of HCT116-CRR xenograft tumors (Scale bar = 200 μm); Right panel: Hi Proportion of Necrotic Area for HCT116-CRR xenografts. Data are presented as mean ± SD (*n* = 3). ^*^P < 0.05, ^**^P < 0.01, ^***^P < 0.001 vs. Control; ^#^*P* < 0.05, ^##^*P* < 0.01 vs. RT + ARE

Microscopic examination of H&E-stained tumor tissues revealed marked cellular atypia in human colorectal cancer xenografts across all groups, characterized by enlarged nuclei with coarse chromatin, prominent nucleoli, frequent mitotic figures, and basophilic cytoplasm. Tumor cells exhibited invasive growth patterns characterized by nested or cord-like arrangements, with focal areas of giant cell formation and central necrotic foci. Notably, control group tumors displayed extensive hemorrhagic necrosis with erythrocyte infiltration in central regions, while all treatment groups showed expanded necrotic areas compared to controls. The most pronounced necrosis was observed in the combination therapy group. To assess the extent of necrosis, ImageJ software was used to analyze the necrotic areas in this study. The results showed that in both HCT116 (*P* < 0.001) and HCT116-CRR (*P* < 0.001) xenografts, the necrotic area was significantly increased in the combination therapy group compared to the control group (Fig. 3A, B). These findings collectively indicated that ARE combined with RT enhanced necrotic cell death in human colorectal cancer xenografts, particularly in combination with radiotherapy.

### EMT in colon cancer

**Fig. 4:**
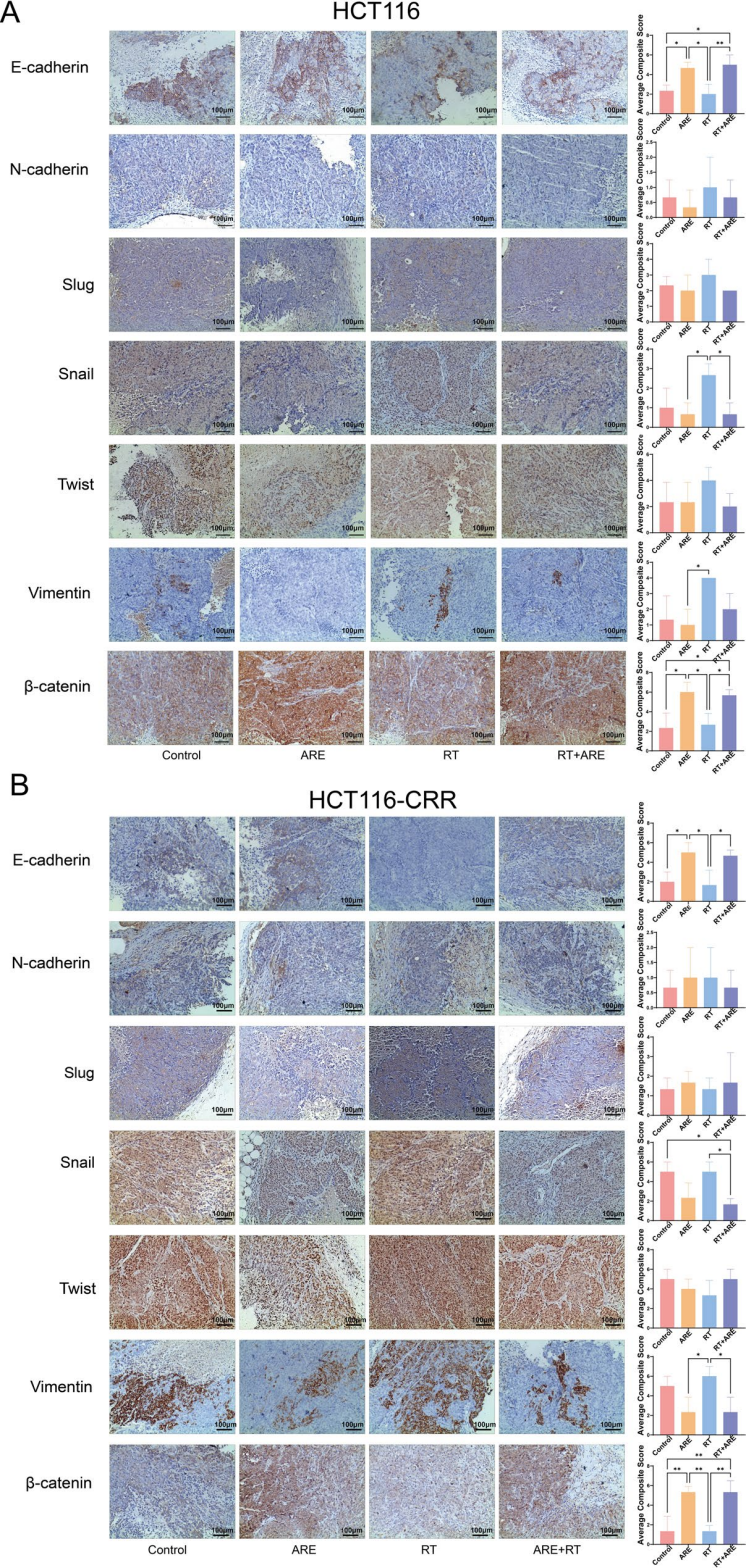
IHC staining of EMT-related markers in HCT116 and HCT116-CRR xenograft tumors. **A** Expression of E-cadherin, N-cadherin, Vimentin, Snail, Slug, Twist, and β-catenin in HCT116 xenografts (Scale bar = 100 μm). **B** Expression of the aforementioned markers in HCT116-CRR xenografts (Scale bar = 100 μm). *P < 0.05, **P < 0.01, ***P < 0.001

Immunohistochemistry results (Fig. 4) revealed that epithelial-mesenchymal transition (EMT)-related markers exhibited treatment-dependent differential expression patterns in xenograft tissues derived from HCT116 and HCT116-CRR colorectal cancer cells. In HCT116 xenografts (Fig. 4A), the epithelial markers E-cadherin and β-catenin exhibited weak positive expression in the control group, while their expression was significantly downregulated in the RT group compared to the ARE group (*P* < 0.05). In contrast, both the ARE and RT + ARE groups showed strong positive expression of these markers, with a noticeable recovery in the combination group compared to the RT group alone. Regarding mesenchymal markers, N-cadherin expression remained negative across all groups. Vimentin, Snail, Slug, and Twist exhibited low expression in the control group, upregulated expression in the RT group, and sustained low expression levels in both the ARE and RT + ARE groups. In HCT116-CRR resistant cell xenografts (Fig. 4B), due to inherent enhanced EMT activity, the epithelial markers E-cadherin and β-catenin showed low expression in the control group, while the mesenchymal markers Vimentin, Snail, and Twist exhibited strong positive expression. Radiotherapy alone led to a further marked upregulation of Vimentin expression. In contrast, both the ARE and RT + ARE groups significantly reversed this trend: E-cadherin and β-catenin were upregulated to strong positive expression, mesenchymal markers such as Vimentin were downregulated to weak positive expression, and N-cadherin remained negative. In summary, ARE could inhibit the EMT process by upregulating epithelial markers and suppressing mesenchymal marker expression; this effect remained stable when combined with radiotherapy and was particularly pronounced in reversing EMT in resistant cells. This provides phenotypic evidence for ARE’s role in enhancing the radiosensitivity of colorectal cancer cells and reversing radio-chemotherapy resistance.

### Expressions of some EMT-related genes

**Fig. 5:**
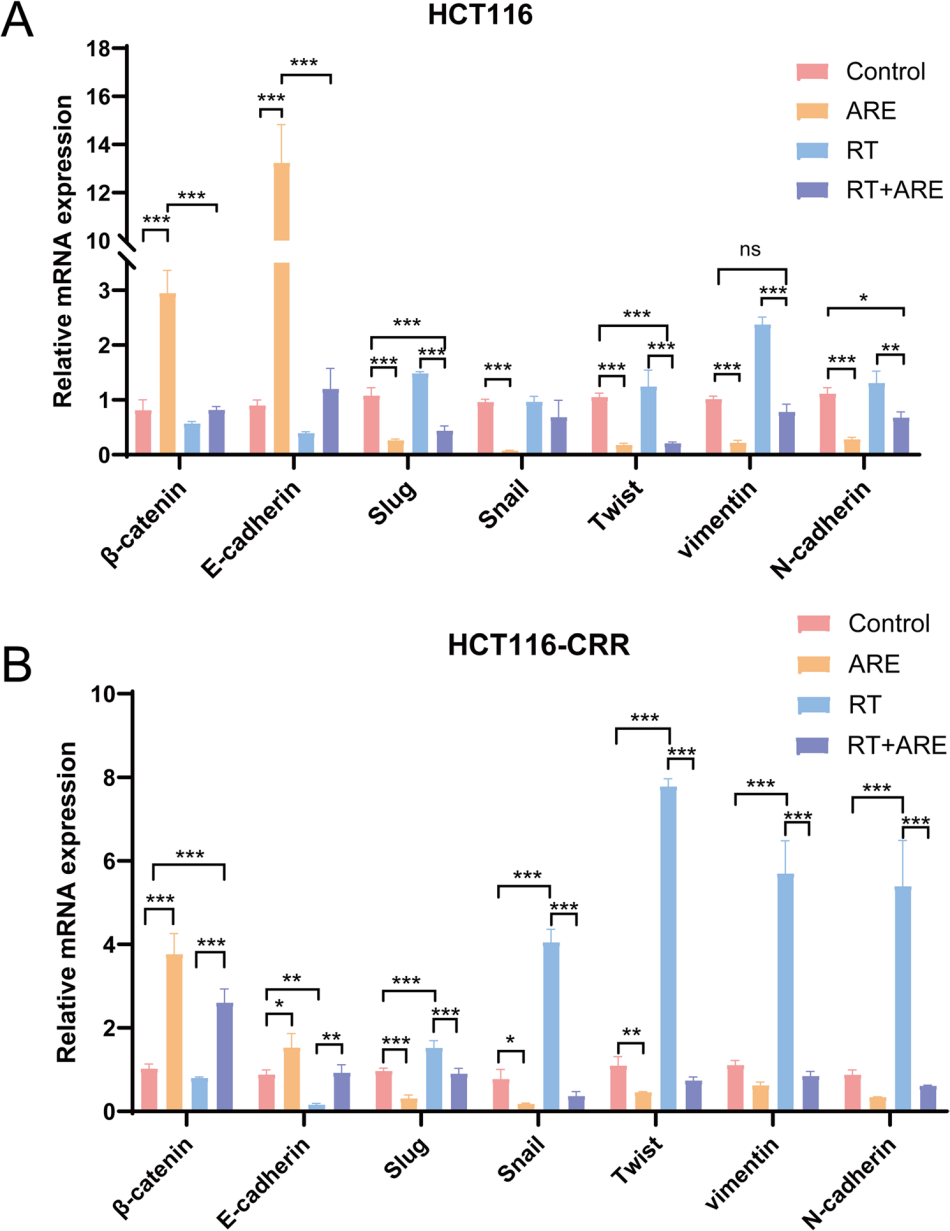
Relative expression of some EMT-related genes in colon cancer. **A** Expressions of EMT-related genes in HCT116. **B** Expressions of EMT-related genes in HCT116-CRR. Data are presented as mean ± SD (*n* = 3). *P < 0.05; ^**^P < 0.01; ^***^P < 0.001

Relative gene expression levels of EMT-related markers (E-Cadherin, N-Cadherin, Vimentin, Snail, Slug, Twist, and β-Catenin) in xenograft tumors of nude mice were analyzed by RT-PCR. Quantitative fluorescence data were analyzed using the 2 ^(−ΔCt)^ method to evaluate inter-group differences in gene expression, with results presented as 2^(−ΔΔCt)^ values. In HCT116 cells (Fig. 5A), compared with the control group, the ARE group exhibited significantly elevated gene expression levels of E-cadherin and β-catenin, whereas the expression levels of N-cadherin, vimentin, Slug, Snail, and Twist were significantly reduced, suggesting that ARE effectively inhibits the EMT process. In contrast, the RT group demonstrated a decreased relative expression level of E-cadherin compared to the control group, while the relative expression levels of Snail, Slug, vimentin, N-cadherin, and Twist were increased, indicating that radiotherapy promotes EMT phenotypic transformation in HCT116 cells. Notably, when compared to the RT group, the combination group showed higher relative expression levels of E-Cadherin and β-Catenin, along with lower relative expression levels of N-Cadherin, Vimentin, Snail, Slug, and Twist in HCT116-CRR cells transplanted into nude mice (Fig. 5B). Compared to the RT group, the combination group demonstrated higher β-Catenin expression and lower Vimentin expression in human colorectal cancer HCT116 cells implanted in nude mice. These results collectively indicated that ARE inhibits EMT in both parental and drug-resistant colorectal cancer cells and can effectively reverse radiotherapy-induced EMT phenotypic transformation, providing molecular-level support for the combined treatment regimen.

### Expression levels of some EMT-related proteins in colon cancer

**Fig. 6:**
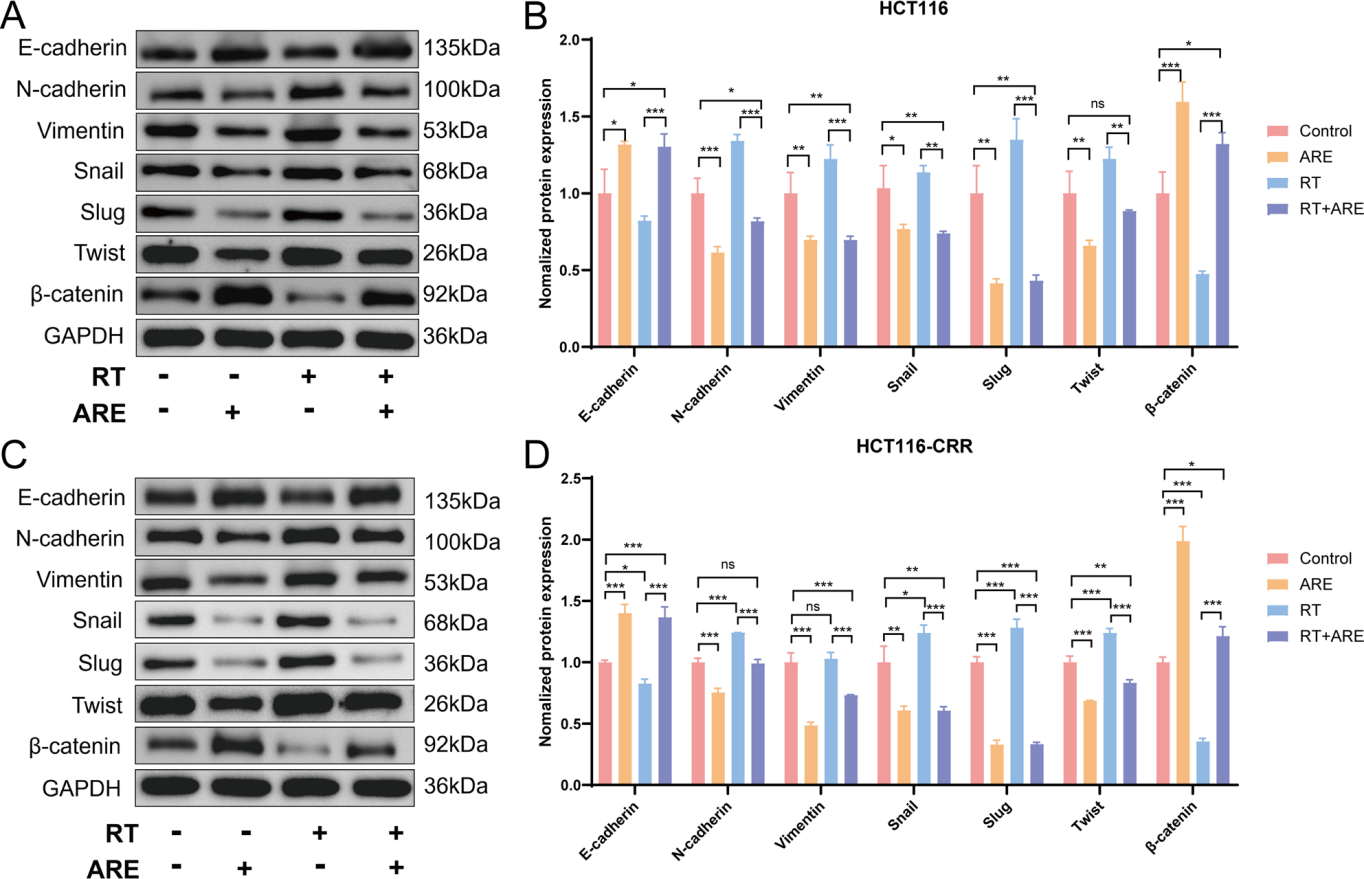
Expression levels of some EMT-related proteins in colon cancer. **A**-**B** Expressions of EMT-related proteins in HCT116. **C**-**D** Expressions of EMT-related proteins in HCT116-CRR. Data are presented as mean ± SD (*n* = 3). *P < 0.05; ^**^P < 0.01; ^***^P < 0.001

Western blot analysis was conducted to evaluate the protein expression levels of EMT-related markers, including E-Cadherin, N-Cadherin, Vimentin, Snail, Slug, Twist, and β-Catenin. Representative protein bands are presented, and the results are expressed as mean ± standard deviation (Fig. 6). In human colorectal cancer xenografts, the ARE group exhibited significantly increased protein expression of E-Cadherin and β-Catenin compared to the control group, whereas the expression levels of N-Cadherin, Vimentin, Slug, and Twist were markedly reduced. The combination therapy group demonstrated enhanced expression of E-Cadherin and β-Catenin and decreased expression of Vimentin and Slug relative to the control group. Compared with the RT group, the combination group showed significantly higher β-Catenin expression and lower Vimentin expression. These findings suggest that the combination of ARE and RT upregulates the epithelial marker β-Catenin and downregulates the mesenchymal marker Vimentin at the protein level in human colorectal cancer xenografts.

## Discussion

The purpose of this study was to verify whether ARE can enhance the radiotherapy efficacy in human colorectal cancer xenografts in nude mice and explore whether its potential mechanism is related to the regulation of EMT. To enhance the consistency and reproducibility of the model, this study employed a two-step modeling procedure involving secondary transplantation of tissue blocks obtained from primary xenograft tumors. Pre-experimental results indicated that tumors established with this method exhibited improved uniformity and stability in size and morphology. This approach helps to achieve structurally more stable and growth-synchronized tumor models prior to treatment, thereby reducing the impact of individual variability on the assessment of therapeutic response. This optimization is particularly important for the evaluation of radiotherapy and other interventions, and does not introduce new genetic heterogeneity (all seed cells were HCT116/HCT116-CRR). To more objectively evaluate potential phenotypic or microenvironmental heterogeneity introduced during the transplantation process and to provide direct evidence for its clinical relevance, subsequent studies will conduct quantitative characterization and validation of the model heterogeneity through corresponding experiments.

By observing the general condition and body weight changes of tumor-bearing mice, comparing the average tumor volume changes, tumor volume inhibition rate, EF, average tumor weight, and tumor inhibition rate, it was demonstrated that ARE itself inhibits the growth of human colorectal cancer xenografts and enhances the radiosensitivity and reverses chemo-radiation resistance of human colorectal cancer xenografts to radiotherapy. It should be noted that in the HCT116 model, although the tumor inhibition levels of the combination therapy group (RT + ARE) and the radiotherapy-alone group (RT) were similar at the endpoint, this may be related to the limitation of single time-point measurement. The core advantage of the combination therapy lies primarily in its significant reversal effect on the chemo-radiation resistant model HCT116-CRR, and in its enhancement of therapeutic efficacy through multiple mechanisms such as promoting apoptosis, increasing necrosis, and inhibiting EMT. Therefore, the local similarity in tumor volume does not negate the value of ARE as a radiosensitizer; this study provides solid mechanistic support for its sensitizing effect from the perspective of reversing resistance and regulating key signaling pathways.

Flow cytometry and HE staining further confirmed that ARE induces apoptosis and necrosis in irradiated tumor cells, thereby increasing radiosensitivity and reversing chemo-radiation resistance. Notably, the results from flow cytometry and H&E staining collectively reveal distinct layers of cell death mechanisms. Flow cytometry demonstrated that the combined treatment primarily induced primary apoptosis in tumor cells, while the extensive necrosis observed via H&E staining represented secondary ischemic necrosis resulting from effective tumor growth suppression by the treatment. These findings are not contradictory; rather, they confirm from the perspectives of cellular events and tissue architecture, respectively, that the combined therapy enhances radiosensitivity and reverses chemoradiation resistance by promoting both apoptosis and secondary necrosis.

EMT delineates the process whereby epithelial cells undergo a phenotypic shift, relinquishing their epithelial characteristics to adopt mesenchymal traits in response to specific physiological or pathological stimuli. EMT is classified into three distinct types, each characterized by its contextual relevance, biological behavior, and molecular signatures [13, 27]. Type III EMT is characterized by neoplastic cells exhibiting EMT traits, which encompass reduced expression of epithelial markers, elevated levels of mesenchymal markers, increased metalloproteinase activity, and a poorly differentiated phenotype, all contributing to enhanced tumor invasiveness and malignancy. The hallmark of EMT is the loss of epithelial polarity and the gain of mesenchymal traits. This process involves several molecular changes: a decrease in epithelial markers like E-cadherin and β-Catenin, an increase in mesenchymal markers such as vimentin and N-cadherin, heightened expression of EMT-related cytokines and transcription factors (including Snail, Slug, Twist, TGF-β), and higher levels of MMP-2, MMP-9, type I collagen, and type III collagen [28]. Notably, the reduction of E-cadherin is a pivotal event in the initiation of EMT [29].

Cadherins are transmembrane glycoproteins that play a crucial role in cell adhesion and are categorized into three types: E-cadherin, P-cadherin, and N-cadherin. E-cadherin is primarily found in epithelial cells and is significantly downregulated during processes like embryonic development, tissue repair, and tumor formation. This downregulation serves as a key indicator of the EMT, facilitating its initiation. In contrast, N-cadherin is predominantly expressed in mesenchymal cells and aids in the migration and metastasis of tumor cells. Studies show that during EMT, E-cadherin levels decrease while N-cadherin levels rise. Vimentin, though considered an EMT marker, has a controversial status; it indicates Type I EMT during gastrulation and Type III EMT in tumors but complicates the identification of Type II EMT due to its expression in tissue repair. β-Catenin has a dual function in EMT: it links cadherins to the cytoskeleton and collaborates with TCF/LEF to regulate genes associated with EMT, particularly Snail1. Therefore, β-Catenin is a significant biomarker in embryonic development, tissue fibrosis, and malignant tumor metastasis [30]. Snail, a zinc finger transcription factor, plays a significant role in regulating various signaling pathways during EMT. It inhibits E-cadherin expression by competitively binding to the E-box in its promoter while promoting vimentin expression, thus facilitating the transition to a mesenchymal phenotype. Consequently, Snail serves as an important biomarker for EMT [31]. Another member of the Snail family, Slug, influences embryonic development and tumor growth by repressing E-cadherin and β-catenin promoters, as well as tight junction proteins like Occludin and ZO-1, which decreases cell adhesion during EMT. In malignant tumor cells undergoing Type III EMT, Twist enhances Snail gene expression and downregulates E-cadherin, establishing itself as a crucial transcription factor marker [32].

Our findings demonstrate that following ARE combined with radiotherapy, the expression of the epithelial marker β-catenin increased, while the mesenchymal marker vimentin decreased in HCT116 xenografts. In HCT116-CRR xenografts, the epithelial markers E-cadherin and β-catenin were up-regulated, whereas the mesenchymal markers N-cadherin, vimentin, Snail, Slug, and Twist were down-regulated. It is noteworthy that β-catenin, as a core transcriptional co-activator of the Wnt signaling pathway, is often associated with tumor progression when its expression increases. This appears to superficially contradict the EMT-inhibitory phenotype observed in this study, highlighting the functional complexity of β-catenin. We propose a reasonable speculation: the upregulated β-catenin may be preferentially recruited to and retained at the cell membrane, where it strengthens E-cadherin-mediated adherens junctions, stabilizes epithelial architecture, and thereby inhibits the EMT process; meanwhile, its nuclear transcriptional activity may play a relatively minor role in this specific context. This “membrane-tropic function” hypothesis coherently explains the concurrent increase in total β-catenin levels and the suppression of the EMT phenotype. Nevertheless, this proposed functional shift requires direct validation in future studies through experiments such as subcellular localization analysis. Clarifying the spatial redistribution of β-catenin following ARE treatment will be key to elucidating its specific mechanism of action in this study. Concurrently, this study also observed certain discrepancies between mRNA and protein expression levels for some EMT markers. Such disparities are common in biological research and may arise from post-transcriptional regulatory mechanisms, such as differences in mRNA stability, microRNA-mediated translational repression, or protein degradation rates. These observations do not affect the core conclusion of this study that ARE inhibits EMT and reverses radiotherapy resistance; rather, they suggest that ARE may influence the EMT process through a multi‑layered regulatory network. Future studies should directly validate the regulation of β‑catenin functional status by ARE through methods such as subcellular localization analysis, and further systematic investigation into protein stability, translation efficiency, and related regulatory molecules (e.g., specific microRNAs) could clarify these post‑transcriptional regulatory mechanisms, thereby providing a more comprehensive elucidation of the molecular landscape underlying its EMT‑reversing effects. These results indicate that ARE may function as an agent capable of reversing EMT in colorectal cancer cells.

Although it was observed that ARE combined with radiotherapy could modulate EMT-related markers and was associated with tumor growth inhibition and radiosensitization, the current results primarily reflect a correlation rather than establishing a direct causal relationship. While EMT reversal may be a key mechanism involved, the participation of other signaling pathways cannot be excluded. Furthermore, the specific upstream molecular mechanisms through which ARE reverses EMT—such as whether it acts by regulating the Wnt/β-catenin, NF-κB, or TGF-β pathways—remain unclear. Direct functional validation through experiments such as cell migration and invasion assays is also lacking, and the immunohistochemical analysis did not include independent positive controls. Although existing literature suggests that artemisinin derivatives may exhibit certain selectivity toward tumor cells [33, 34], their systemic safety and therapeutic window still require further clarification through comprehensive toxicity assessments and comparisons with normal cells. The present study primarily provides experimental evidence through in vivo xenograft models for the radiosensitizing effect and EMT regulatory role of ARE. To further distinguish the direct cellular effects of ARE from the overall responses mediated by the tumor microenvironment and to systematically elucidate its mechanism of action, subsequent studies will be conducted in cell culture systems, focusing on the direct effects of ARE on EMT marker expression, cell migration and invasion, and radiosensitivity. Future research should validate these findings in models more closely resembling clinical settings and systematically elucidate the complete pathway by which ARE reverses EMT through functional experiments and mechanistic exploration.

## Conclusions

In summary, this study demonstrates that ARE) a derivative of artemisinin, functions as an effective radiosensitizer in colorectal cancer. Using xenograft models of both parental and chemo-radioresistant CRC cells, we found that ARE combined with radiotherapy not only potently inhibited tumor growth but also reversed acquired resistance. Mechanistically, the radiosensitizing effect of ARE is closely associated with the suppression of epithelial-mesenchymal transition (EMT), as evidenced by the restoration of epithelial markers (E-cadherin, β-catenin) and downregulation of key mesenchymal markers and transcription factors (N-cadherin, Vimentin, Snail, Slug, Twist). These findings provide the experimental basis for further research into artemether as a potential radiosensitizer in colorectal cancer.

## Supplementary Information

Supplementary Material 1.

## Data Availability

All data generated or analysed during this study are included in this article. Further enquiries can be directed to the corresponding author.
