# Supplementary material for: Artemether as a modulator of EMT in colorectal cancer: enhancing radiosensitivity and reversing chemo-radiation resistance
Source: BMC Gastroenterol. 2026 Feb 4;26:157. doi: 10.1186/s12876-026-04653-4 (PMC12964608; doi:10.1186/s12876-026-04653-4)

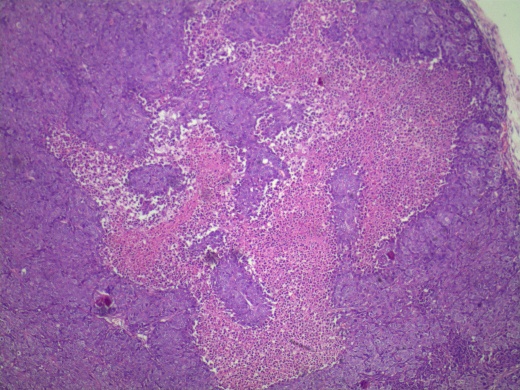


Figure3-A-Control


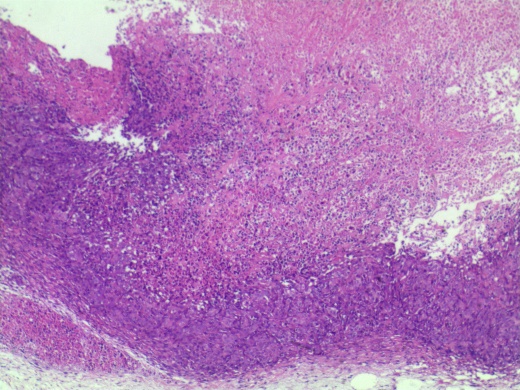


Figure3-A-ARE


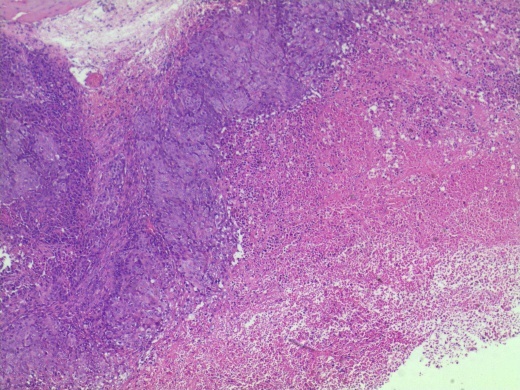


Figure3-A-RT


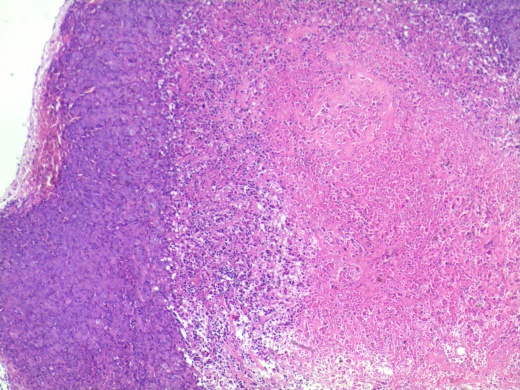


Figure3-A-RT+ARE


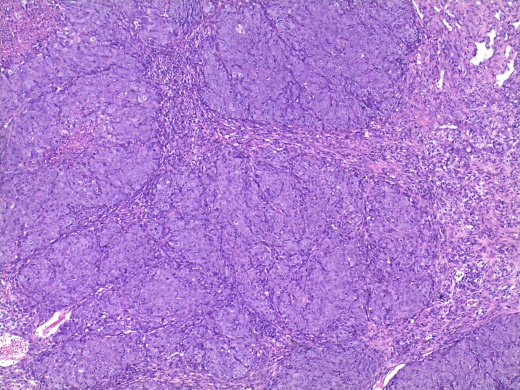


Figure3-B-Control


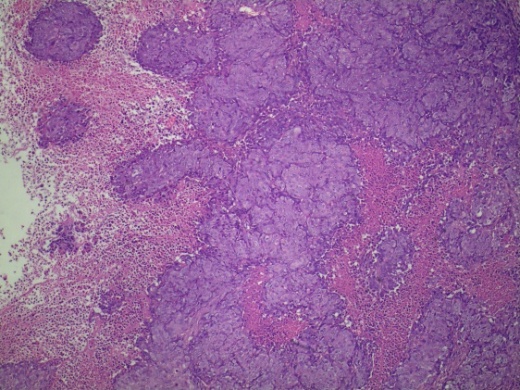


Figure3-B-ARE


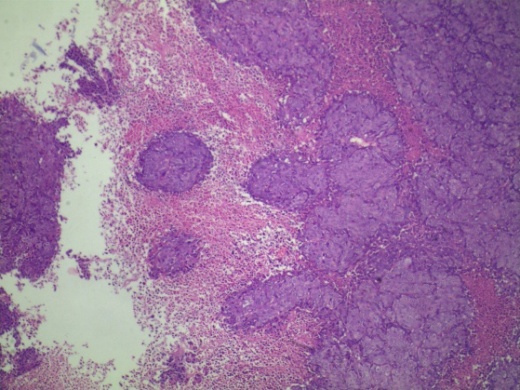


Figure3-B-RT


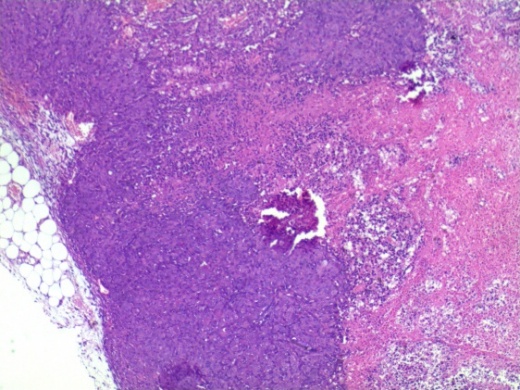


Figure3-B-RT+ARE


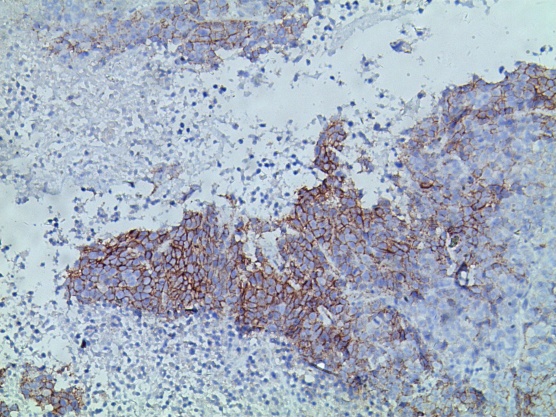


Figure4-A-E-Cadherin-Control


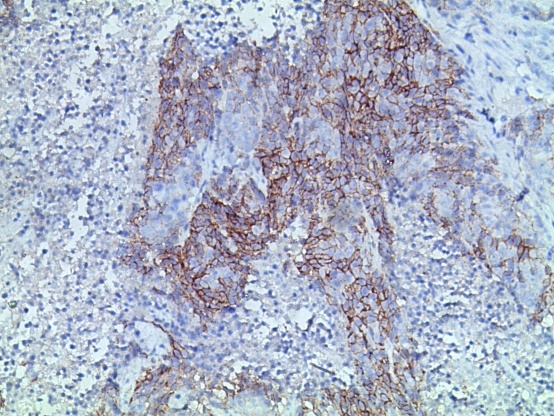


Figure4-A-E-Cadherin-ARE


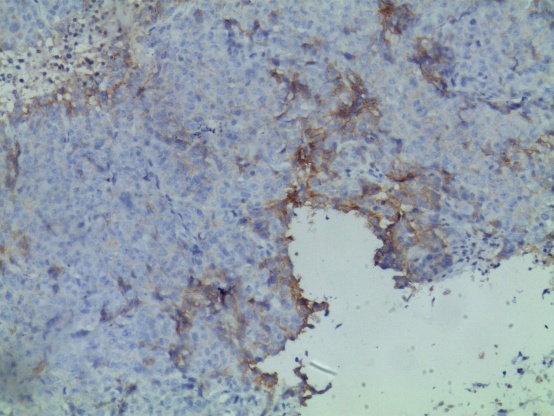


Figure4-A-E-Cadherin-RT


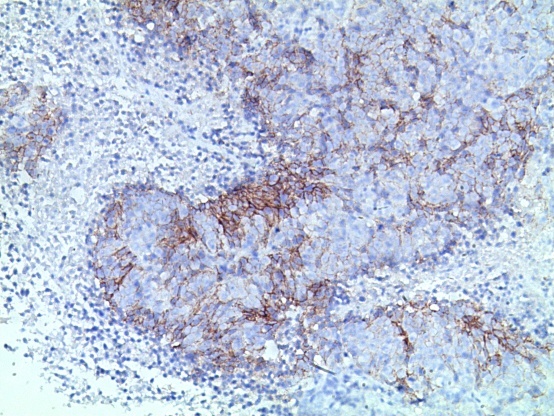


Figure4-A-E-Cadherin-RT+ARE


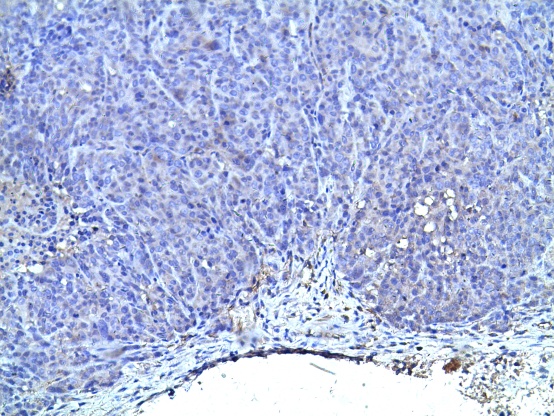


Figure4-A-N-Cadherin-Control


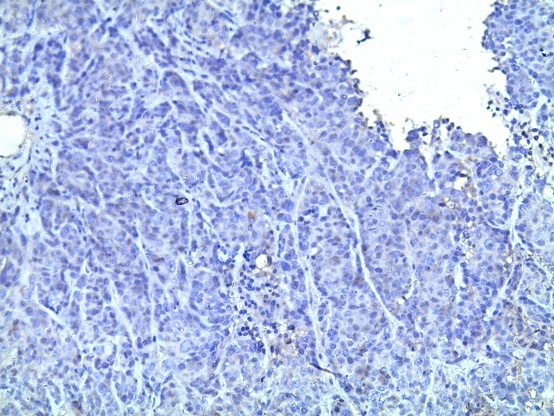


Figure4-A-N-Cadherin-ARE


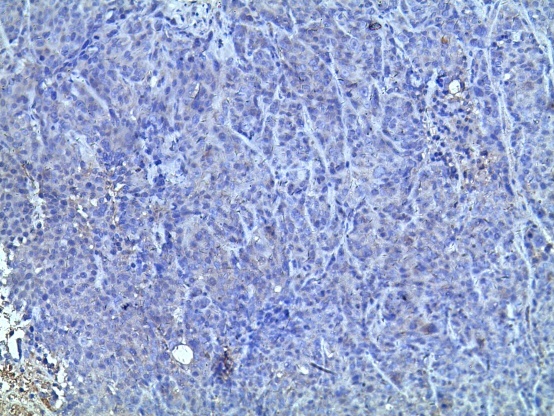


Figure4-A-N-Cadherin-RT


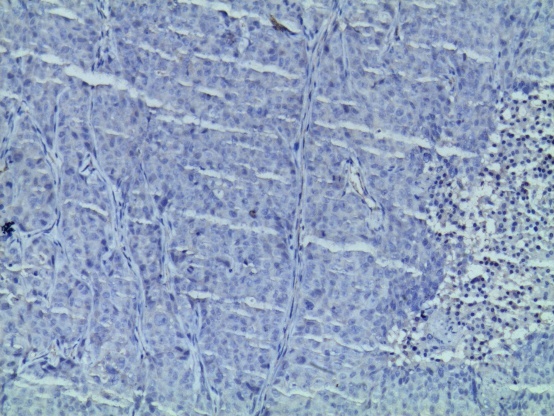


Figure4-A-N-Cadherin-RT+ARE


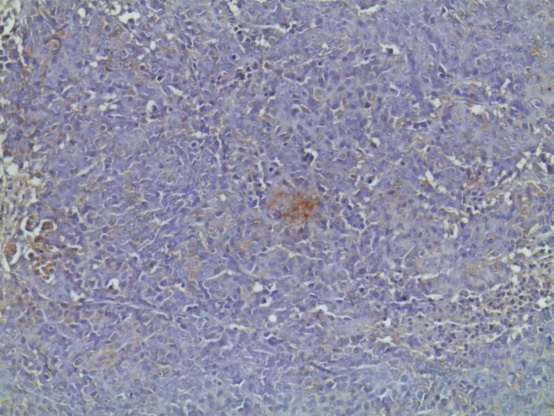


Figure4-A-Slug-Control


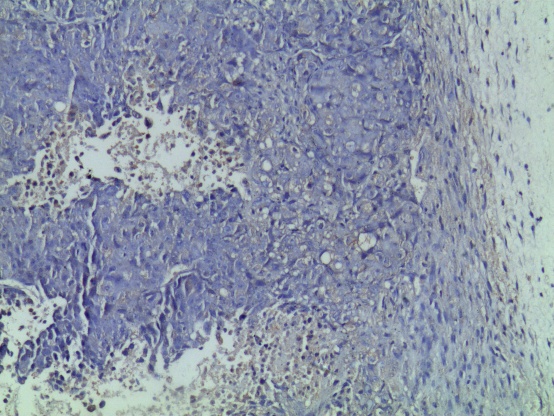


Figure4-A-Slug-ARE


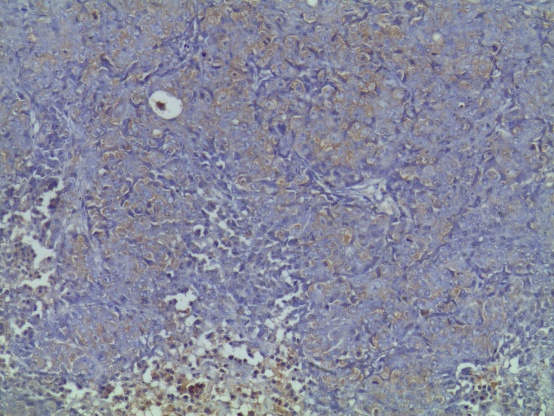


Figure4-A-Slug-RT


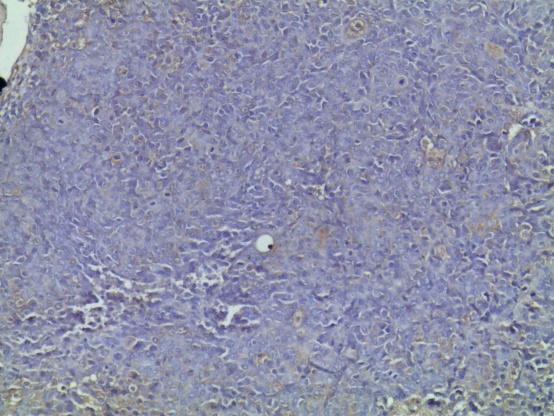


Figure4-A-Slug-RT+ARE


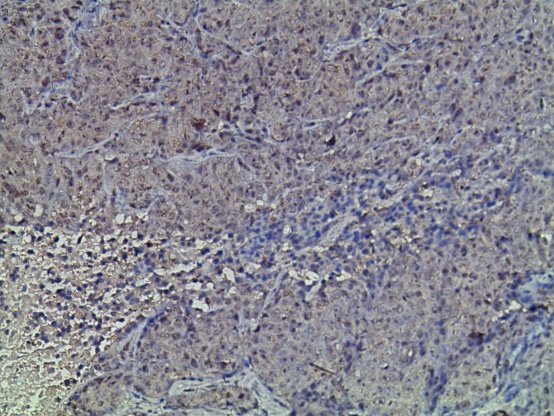


Figure4-A-Snail-Control


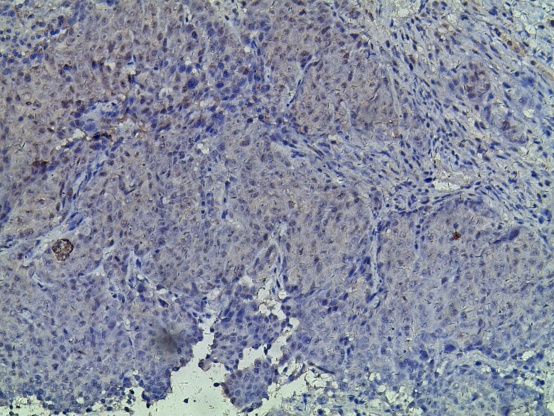


Figure4-A-Snail-ARE


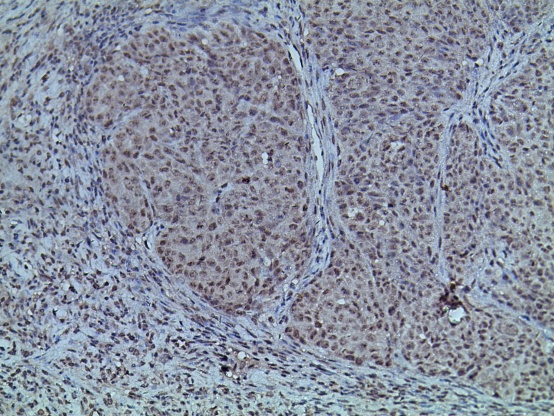


Figure4-A-Snail-RT


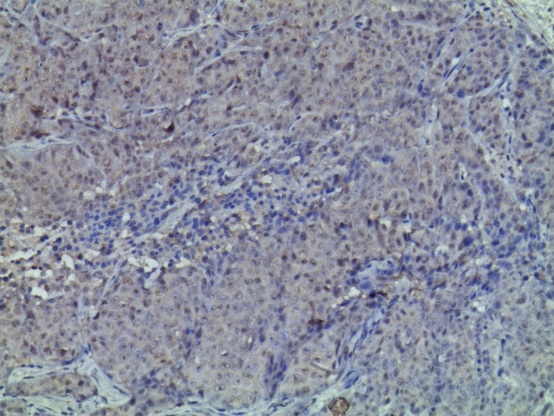


Figure4-A-Snail-RT+ARE


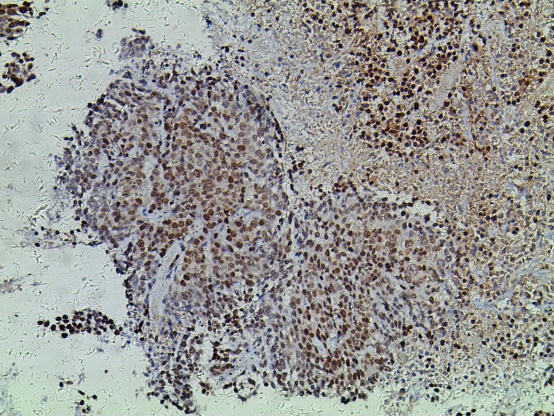


Figure4-A-Twist-Control


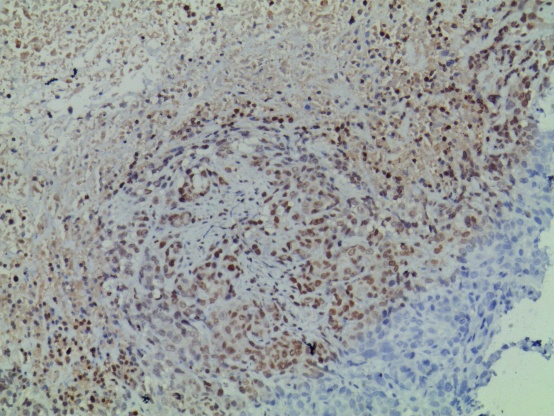


Figure4-A-Twist-ARE


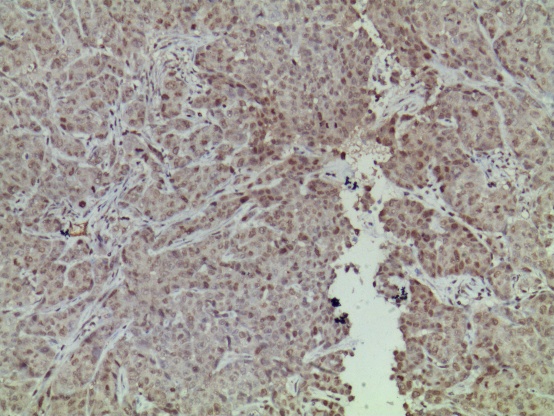


Figure4-A-Twist-RT


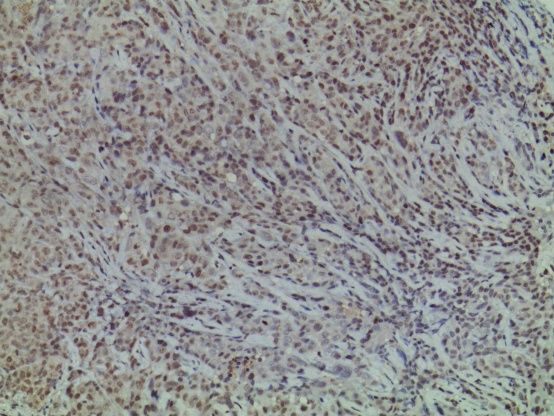


Figure4-A-Twist-RT+ARE


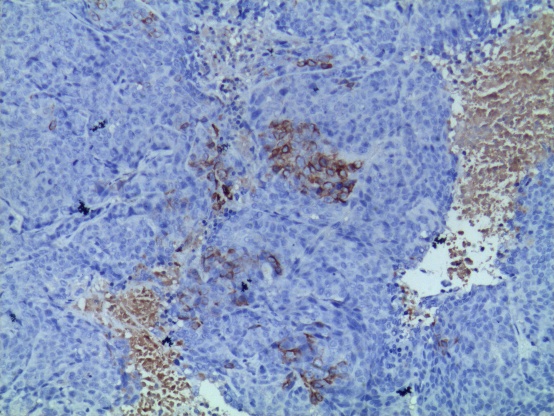


Figure4-A-Vimentin-Control


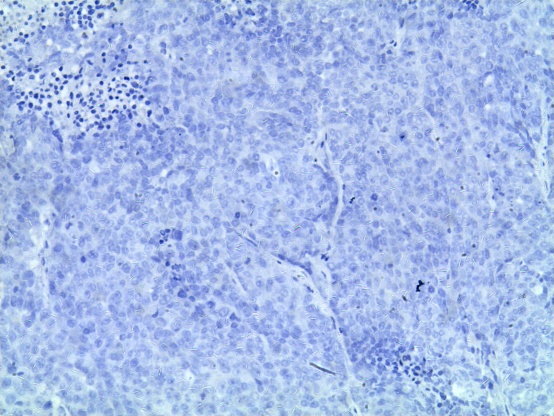


Figure4-A-Vimentin-ARE


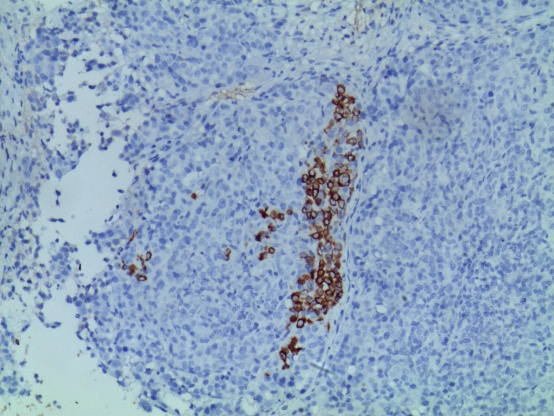


Figure4-A-Vimentin-RT


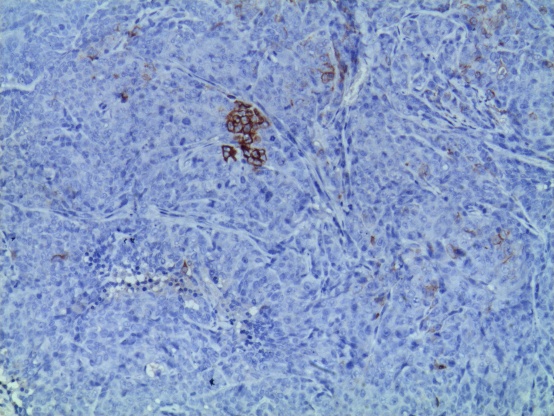


Figure4-A-Vimentin-RT+ARE


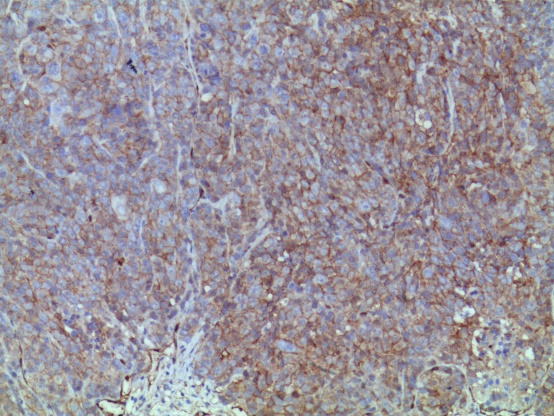


Figure4-A-β-catenin-Control


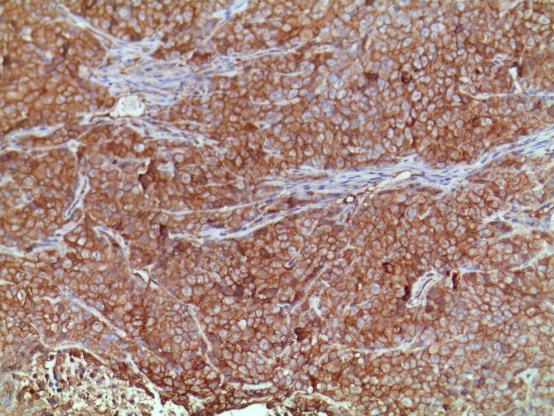


Figure4-A-β-catenin-ARE


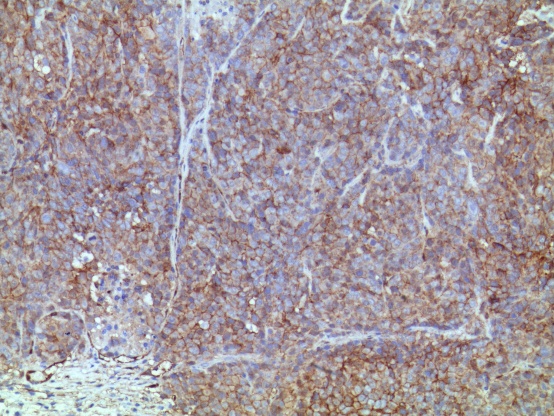


Figure4-A-β-catenin-RT


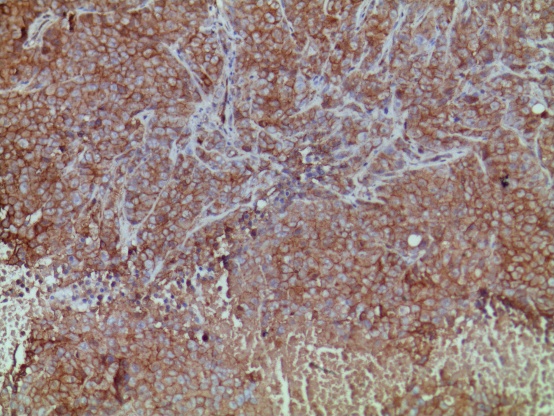


Figure4-A-β-catenin-RT+ARE


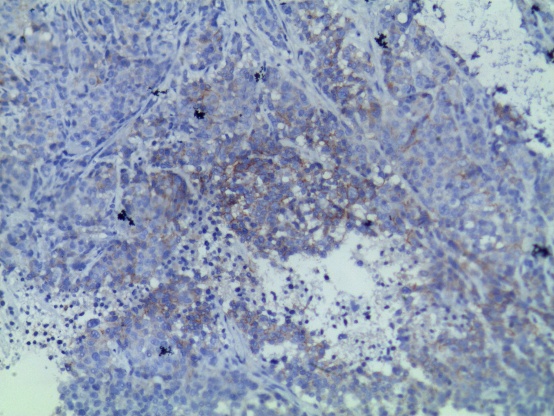


Figure4-B-E-Cadherin-Control


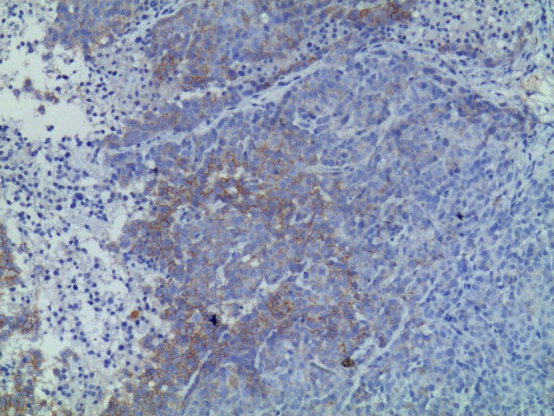


Figure4-B-E-Cadherin-ARE


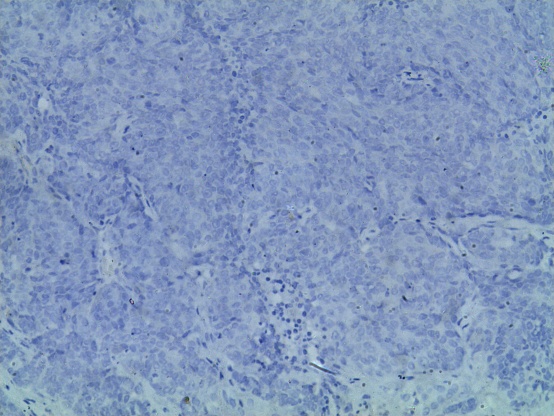


Figure4-B-E-Cadherin-RT


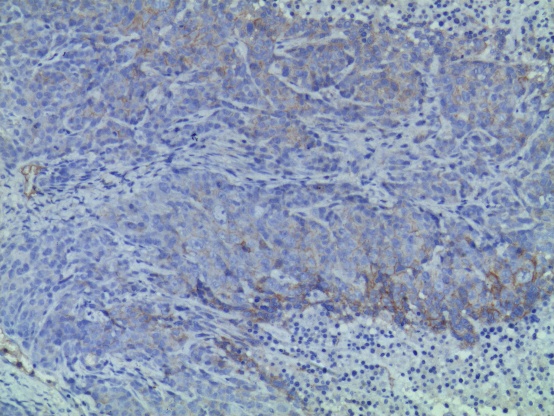


Figure4-B-E-Cadherin-RT+ARE


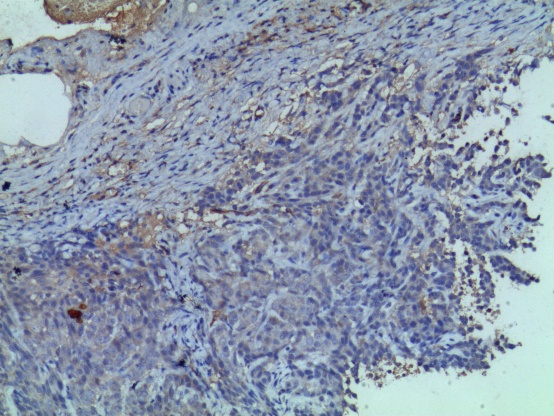


Figure4-B-N-Cadherin-Control


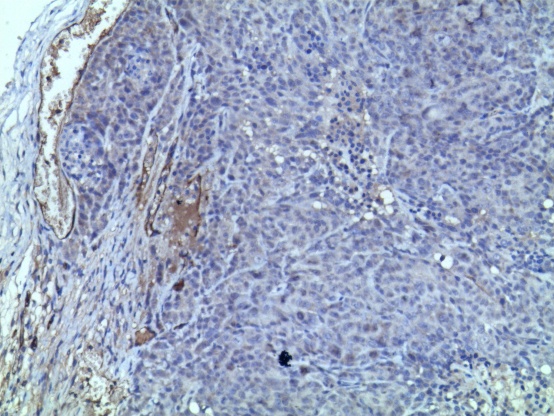


Figure4-B-N-Cadherin-ARE


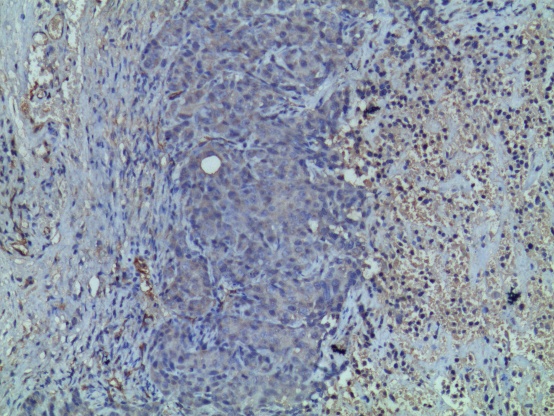


Figure4-B-N-Cadherin-RT


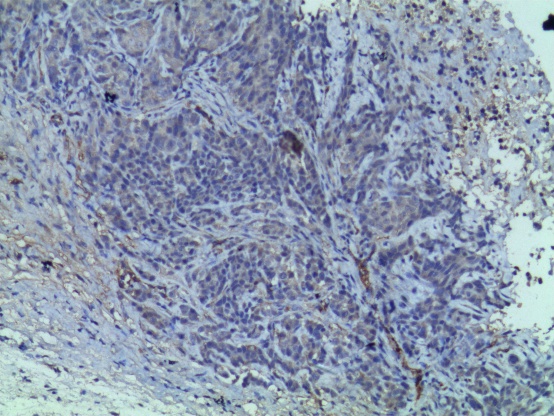


Figure4-B-N-Cadherin-RT+ARE


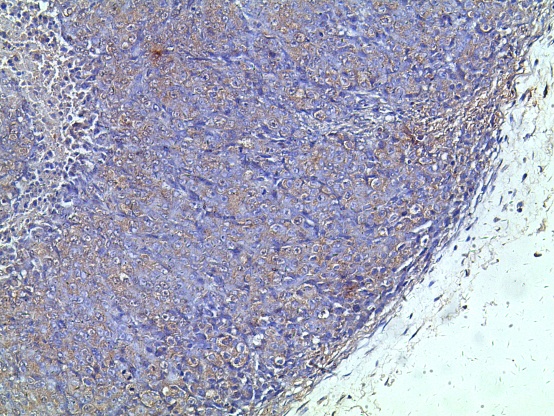


Figure4-B-Slug-Control


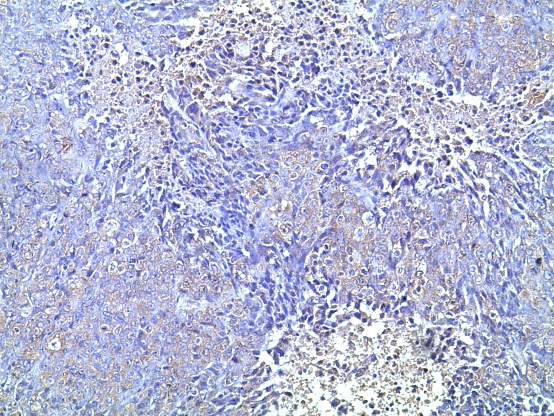


Figure4-B-Slug-ARE


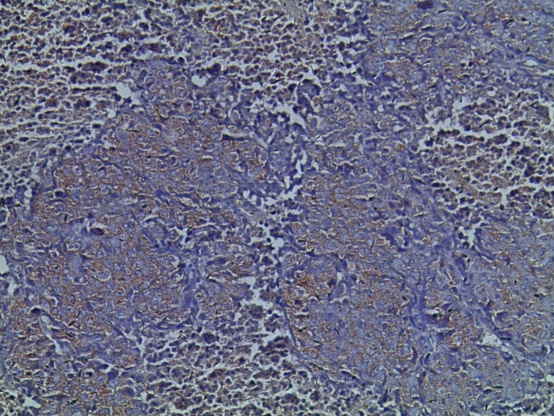


Figure4-B-Slug-RT


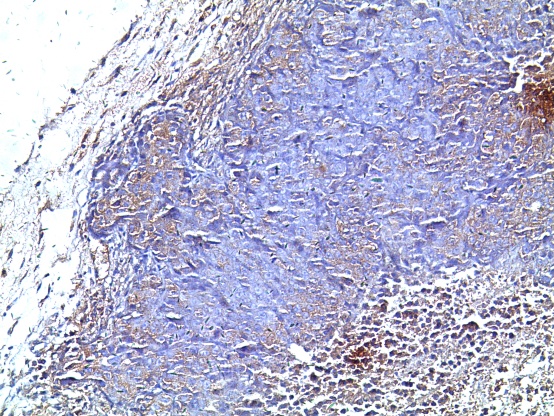


Figure4-B-Slug-RT+ARE


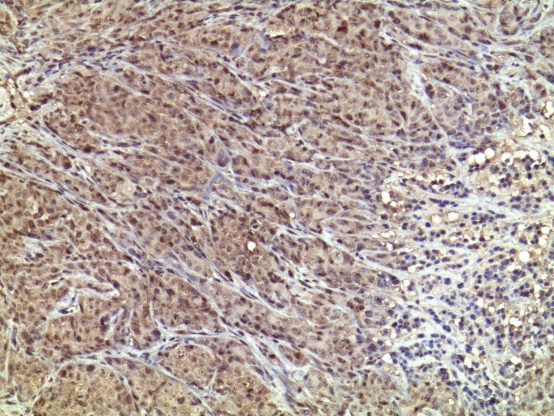


Figure4-B-Snail-Control


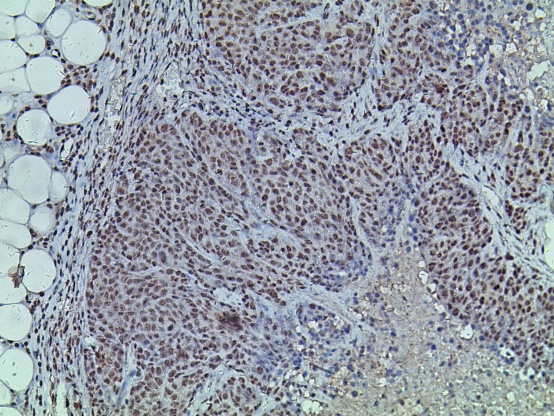


Figure4-B-Snail-ARE


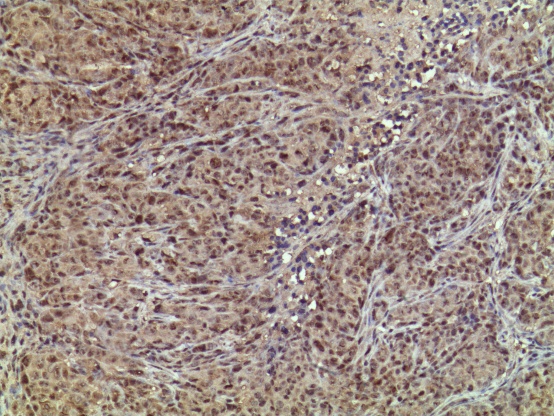


Figure4-B-Snail-RT


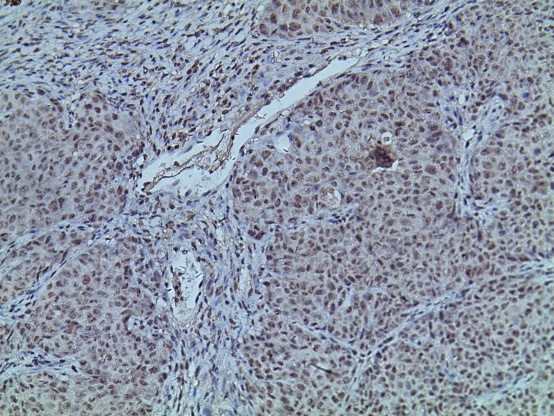


Figure4-B-Snail-RT+ARE


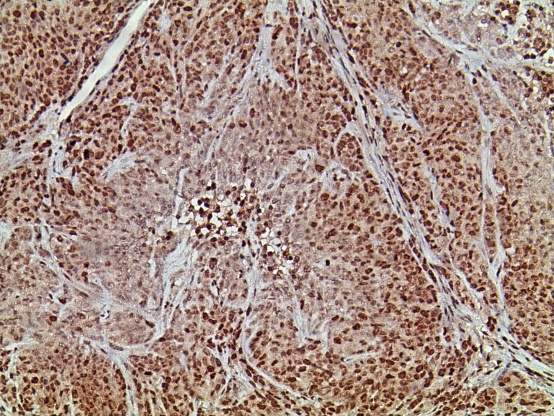


Figure4-B-Twist-Control


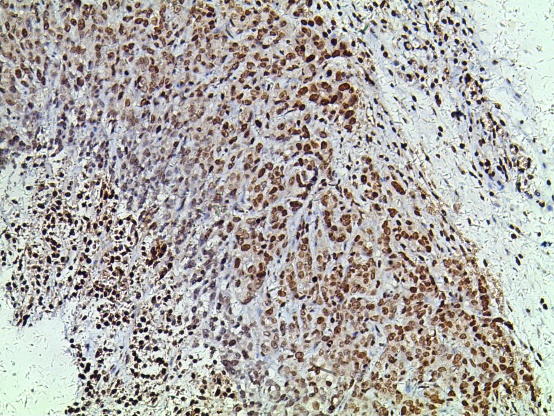


Figure4-B-Twist-ARE


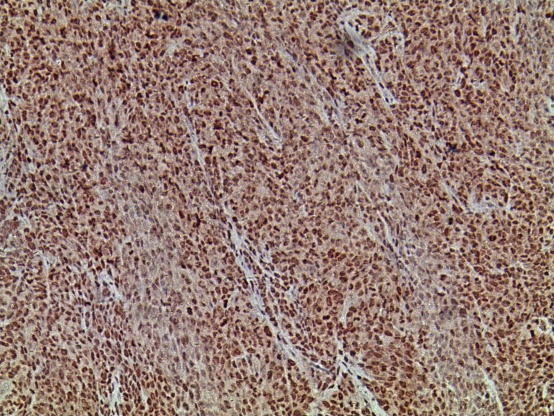


Figure4-B-Twist-RT


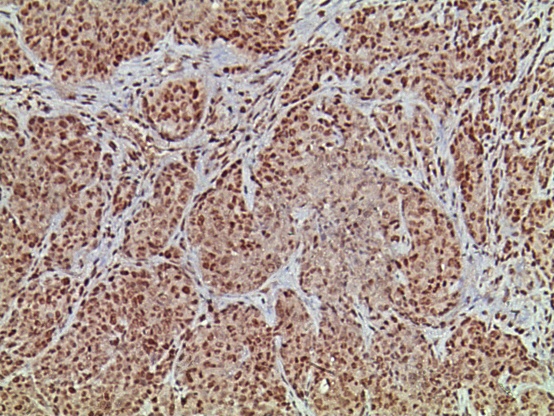


Figure4-B-Twist-RT+ARE


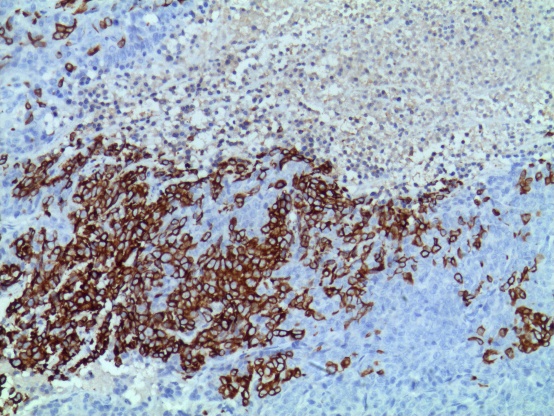


Figure4-B-Vimentin-Control


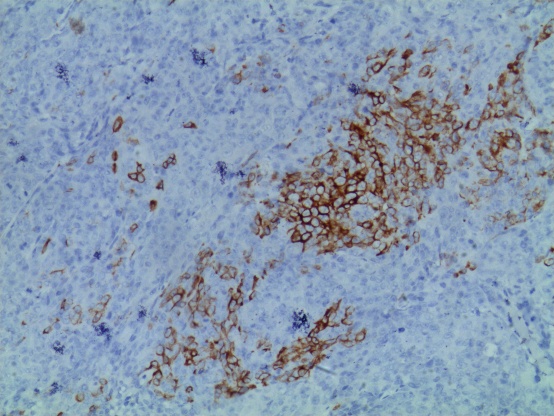


Figure4-B-Vimentin-ARE


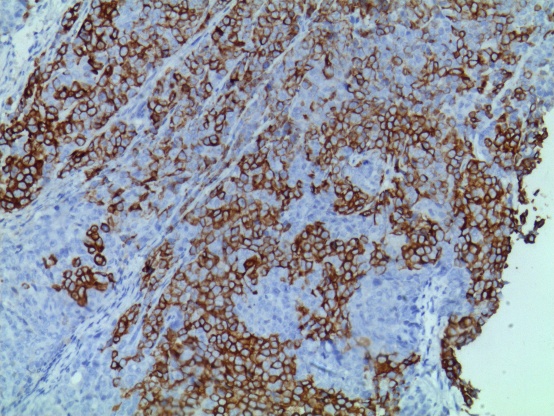


Figure4-B-Vimentin-RT


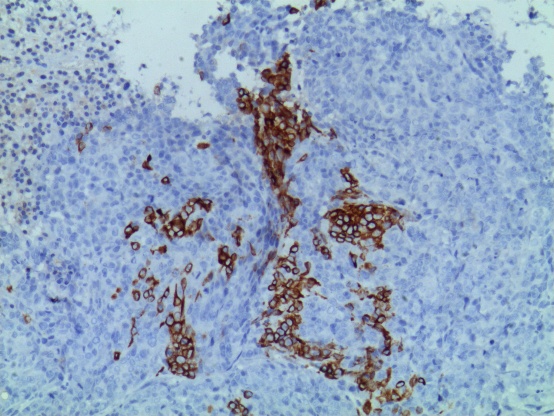


Figure4-B-Vimentin-RT+ARE


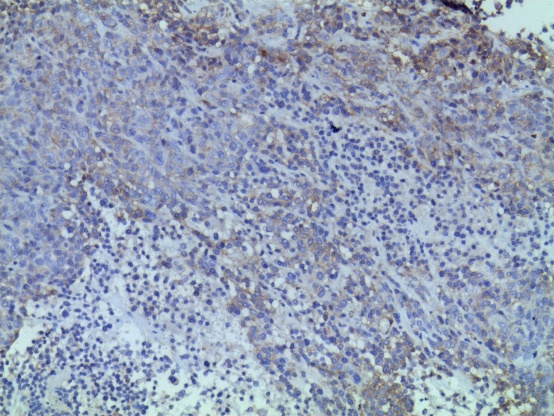


Figure4-B-β-catenin-Control


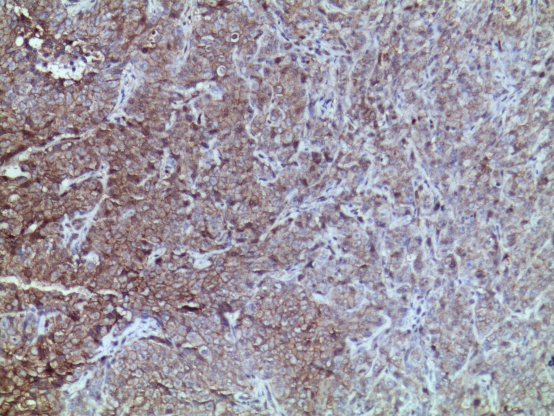


Figure4-B-β-catenin-ARE


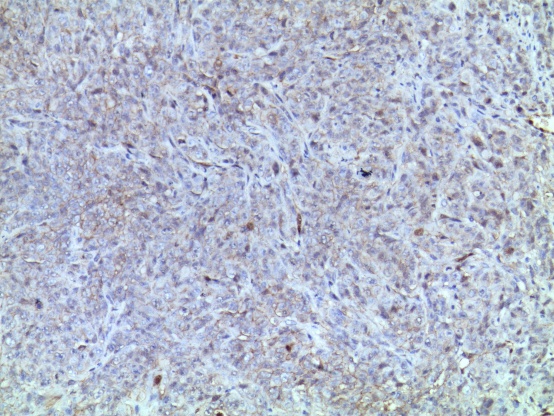


Figure4-B-β-catenin-RT


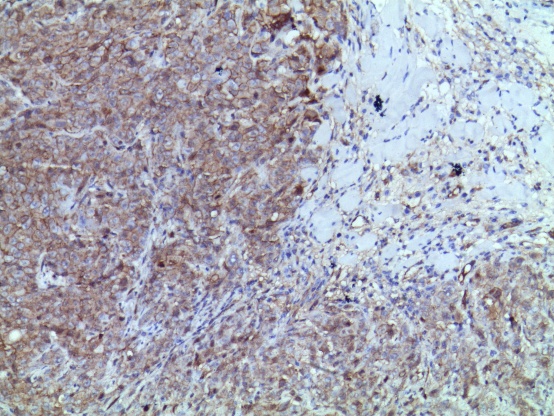


Figure4-B-β-catenin-RT+ARE

Figure 6 A Original Western Blot Image


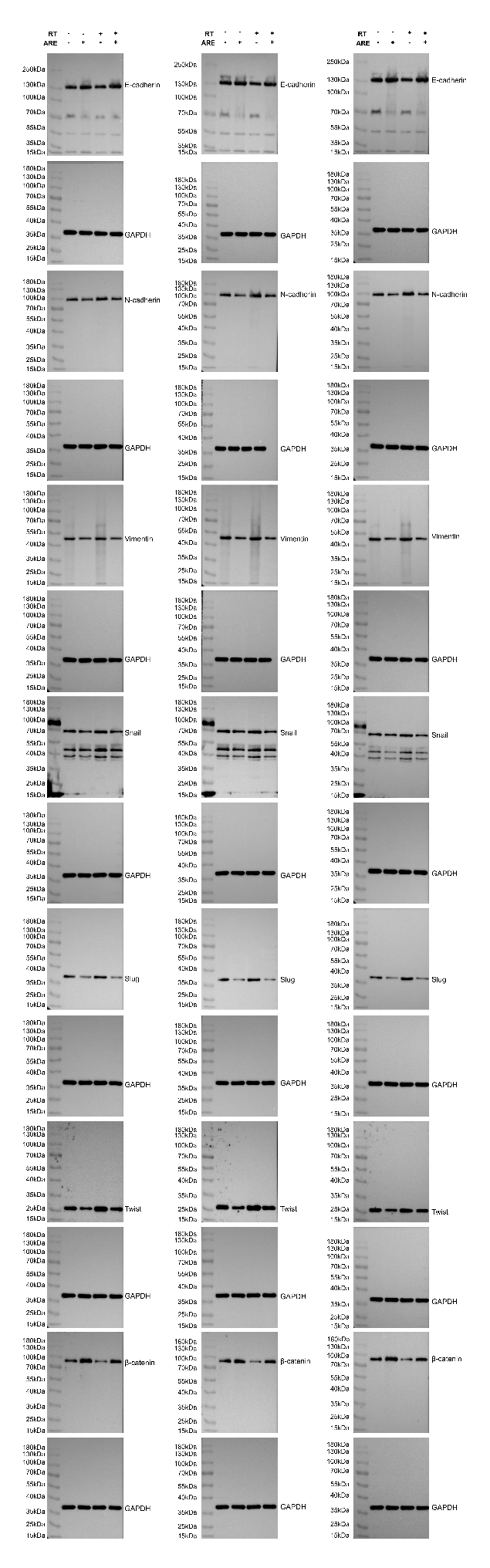


Figure 6 C Original Western Blot Image


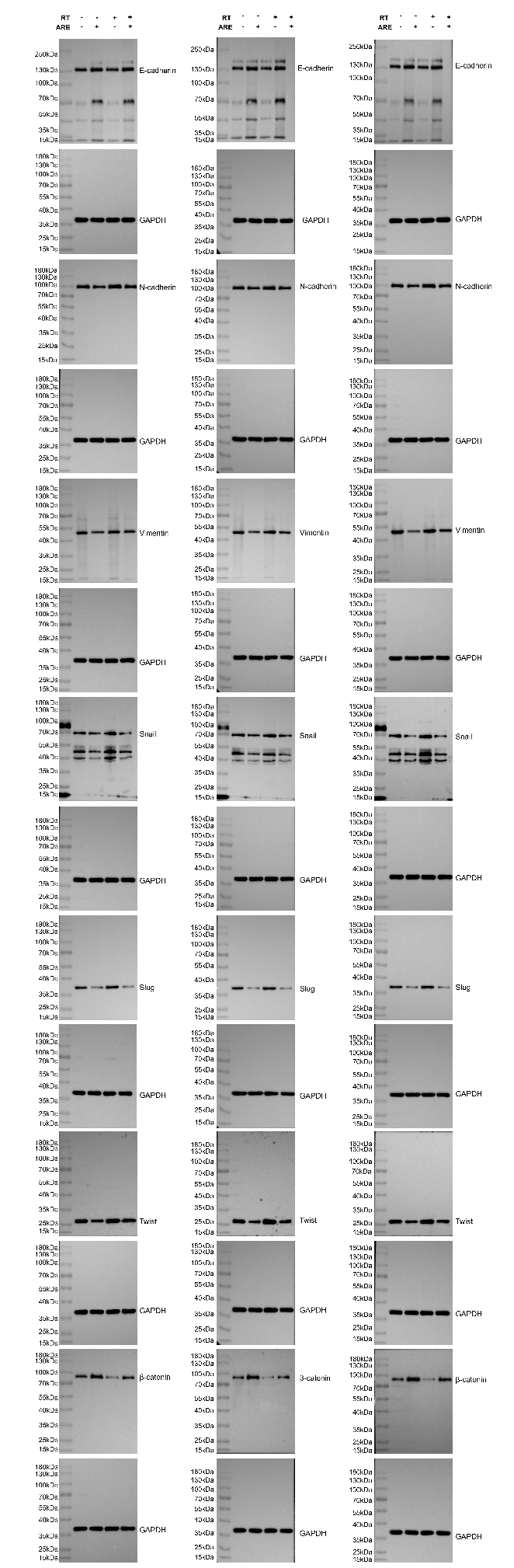


Ethical Approval 1-1


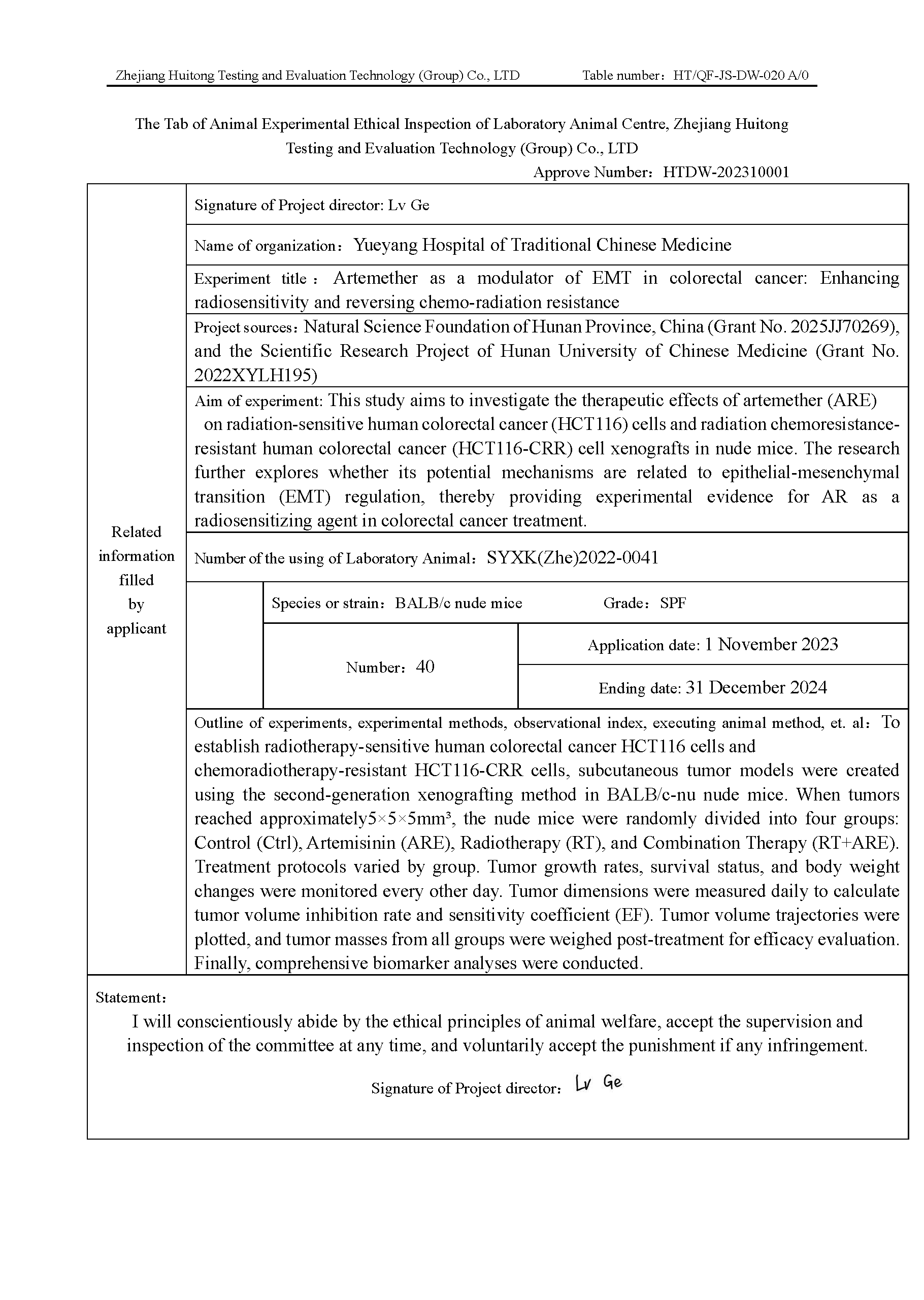


Ethical Approval 1-2


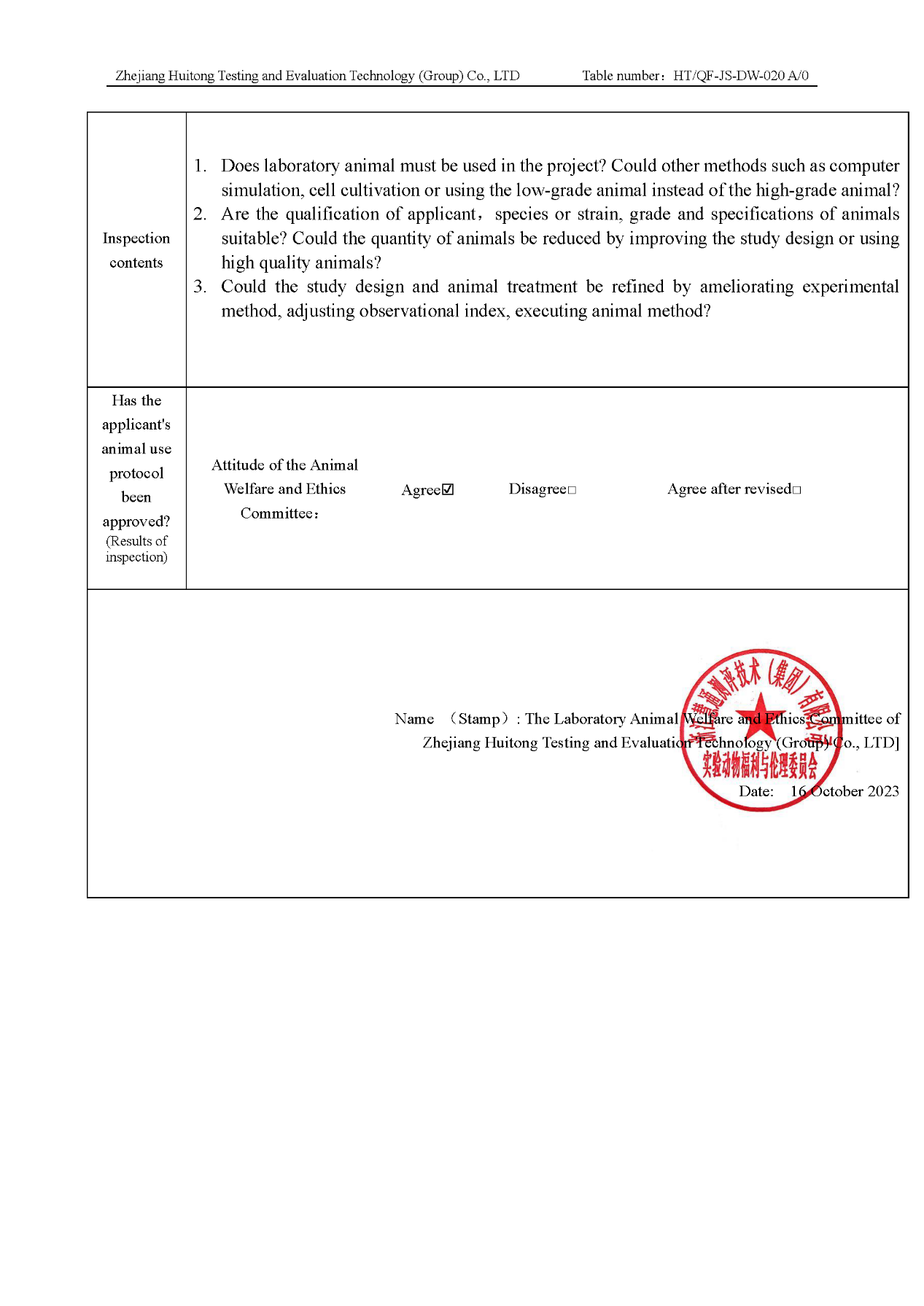

Supplement: Supplementary file 1 — Supplementary Material 1. [file 12876_2026_4653_MOESM1_ESM.docx]
